# CdsA is involved in biosynthesis of glycolipid MPIase essential for membrane protein integration *in vivo*

**DOI:** 10.1038/s41598-018-37809-8

**Published:** 2019-02-04

**Authors:** Katsuhiro Sawasato, Ryo Sato, Hanako Nishikawa, Naoki Iimura, Yuki Kamemoto, Kohki Fujikawa, Toshiyuki Yamaguchi, Yutetsu Kuruma, Yasushi Tamura, Toshiya Endo, Takuya Ueda, Keiko Shimamoto, Ken-ichi Nishiyama

**Affiliations:** 1 The United Graduate School of Agricultural Sciences, Iwate University, Morioka, Iwate 020-8550 Japan; 2 Cryobiofrontier Research Center, Faculty of Agriculture, Iwate University, Morioka, Iwate 020-8550 Japan; 3 Bioorganic Research Institute, Suntory Foundation for Life Sciences, Seika-cho, Kyoto 619-0284 Japan; 4 Earth-Life Science Institute, Tokyo Institute of Technology, Meguro-ku, Tokyo 152-8550 Japan; 5 Faculty of Science, Yamagata University, Yamagata, Yamagata 990-8560 Japan; 6 Faculty of Life Sciences, Kyoto Sangyo University, Kita-ku, Kyoto 603-8555 Japan; 7 Department of Computational Biology and Medical Sciences Graduate School of Frontier Sciences, The University of Tokyo, Kashiwa, Chiba 277-8562 Japan; 8 Department of Biological Chemistry and Food Science, Faculty of Agriculture, Iwate University, Morioka, Iwate 020-8550 Japan

## Abstract

MPIase is a glycolipid that is involved in membrane protein integration. Despite evaluation of its functions *in vitro*, the lack of information on MPIase biosynthesis hampered verification of its involvement *in vivo*. In this study, we found that depletion of CdsA, a CDP-diacylglycerol synthase, caused not only a defect in phospholipid biosynthesis but also MPIase depletion with accumulation of the precursors of both membrane protein M13 coat protein and secretory protein OmpA. Yeast Tam41p, a mitochondrial CDP-diacylglycerol synthase, suppressed the defect in phospholipid biosynthesis, but restored neither MPIase biosynthesis, precursor processing, nor cell growth, indicating that MPIase is essential for membrane protein integration and therefore for cell growth. Consistently, we observed a severe defect in protein integration into MPIase-depleted membrane vesicles *in vitro*. Thus, the function of MPIase as a factor involved in protein integration was proven *in vivo* as well as *in vitro*. Moreover, Cds1p, a eukaryotic CdsA homologue, showed a potential for MPIase biosynthesis. From these results, we speculate the presence of a eukaryotic MPIase homologue.

## Introduction

**Figure 1:**
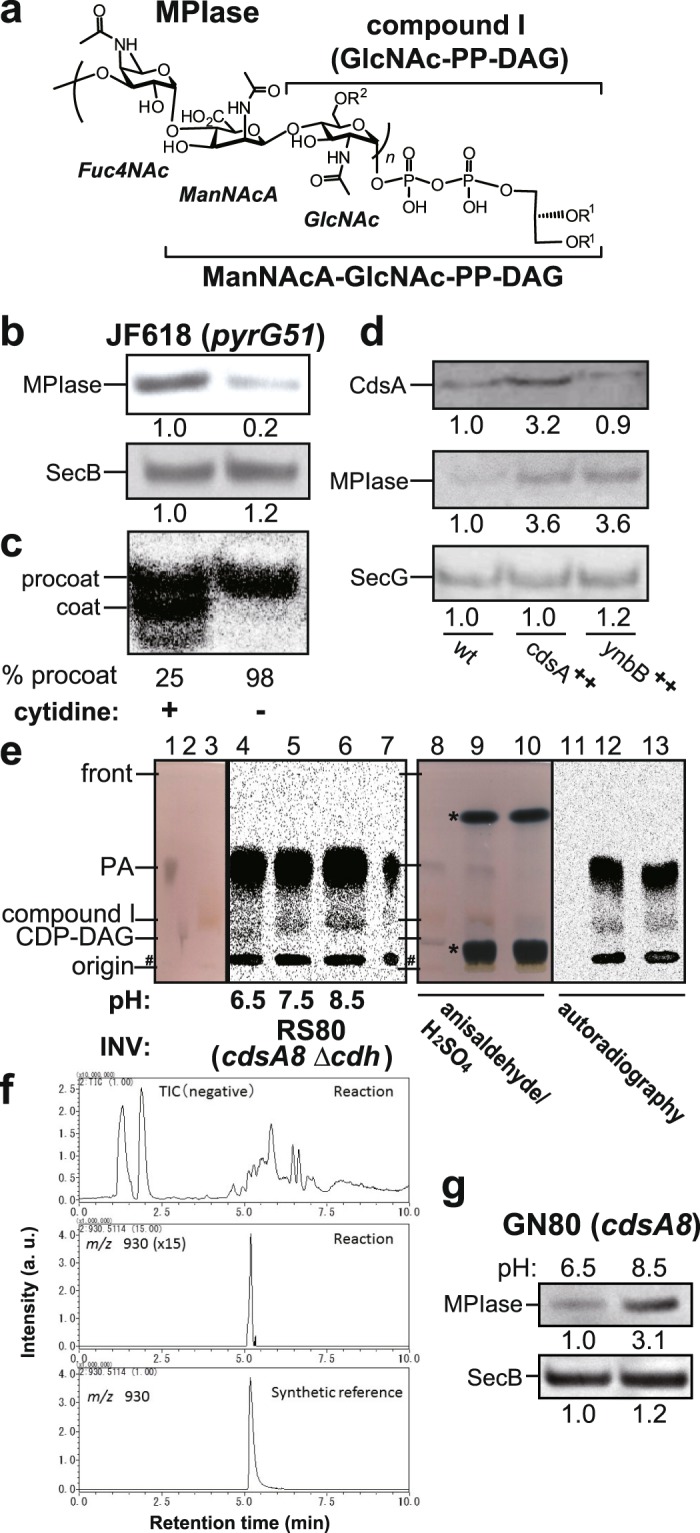
CdsA and YnbB are biosynthetic enzymes for MPIase. (**a**) Structure of MPIase^7^. The parts corresponding to compound I (GlcNAc-PP-DAG) and ManNAcA-GlcNAc-PP-DAG are indicated. (**b**,**c**) Cytidine deprivation causes MPIase depletion (**b**) and accumulation of M13 procoat (**c**). JF618 (*pyrG*) cells, cultivated in M9 medium supplemented with 250 μg/ml cytidine, were washed three times using fresh M9 medium, followed by inoculation into M9 medium supplemented with 250 μg/ml cytidine (+) or not supplemented (−). At the mid log phase, cells were recovered to analyze the MPIase level by SDS-PAGE/immunoblotting. The SecB level was analyzed as a loading control. To examine the processing of M13 procoat, JF618 harboring pMS119-PC was cultivated as in (**b**), followed by IPTG (1 mM) induction. Cells were pulse-labeled for 2 min. M13 procoat and coat were immunoprecipitated and visualized by SDS-PAGE/autoradiography. (**c**,**d**) Overproduction of CdsA and YnbB causes an increase in the MPIase level. INV were prepared from BL21 (wt), BL21 harboring pT7-CdsA (*cdsA*^++^), or BL21 harboring pT7-YnbB (*ynbB*^++^). The levels of CdsA (top), MPIase (middle), and SecG (bottom; loading control) were determined by SDS-PAGE/immunoblotting. The relative amounts of the bands are shown. (**e**) Compound I biosynthesis on the CdsA8 mutant. INV prepared from RS80 (*cdsA8 Δcdh*) were incubated with [^14^C]PA, CTP and GlcNAc-phosphate under the specified conditions, followed by TLC analysis. Synthetic references of PA (lane 1), CDP-DAG (lane 2), and compound I (lane 3) were also analyzed. In lane 7, [^14^C]PA was applied. The references and the sample in lane 6 were co-developed (lanes 9 and 12), while references (lanes 8 and 11) and the sample in lane 6 (lanes 10 and 13) were independently developed, respectively. The spots were visualized with anisaldehyde/H_2_SO_4_ (lanes 8–10) or by autoradiography (lanes 11–13). The contaminants in [^14^C]PA, and are indicated by ‘#’. OG (upper) and sucrose (lower) are by asterisks. (**f**) LC-MS analysis of *in vitro* synthesized compound I. (upper) The total ion chromatogram (TIC) from the biosynthetic reaction mixture. (middle) The extracted ion chromatogram (EIC) of *m/z* 930.5114 from the biosynthetic reaction mixture. (bottom) EIC of *m/z* 930.5114 from the synthetic reference. (**g**) The MPIase level in GN80 (*cdsA8*) increased under non-permissive pH conditions. GN80 grown at pH6.5 was cultivated either at pH6.5 or pH8.5. The levels of MPIase and SecB were determined by SDS-PAGE/immunoblotting.

Protein integration into biological membranes is a vital process in all organisms. The molecular mechanisms underlying protein integration are widely conserved, as the main components involved in protein integration, such as SRP (signal recognition particle), SR (SRP receptor), SecYEG/Sec 61 complex and YidC/Oxa1p/Alb3p, are known to exist ubiquitously. In addition to protein integration that depends on these factors (Sec-dependent integration), a Sec-independent pathway is also known^1–4^. To clarify these mechanisms in detail, we have tried to reconstitute protein integration in a model organism, *E. coli*. We have been successful, the integration reactions *in vivo* being faithfully reflected, and have identified a novel integration factor, glycolipid MPIase, since our structure determination studies revealed that MPIase is a glycolipid devoid of a proteinaceous moiety^5–7^. Thus, MPIase (Fig. 1a) is involved in both Sec-dependent and -independent protein integration into (proteo)liposomes^5–8^. Consistently, an anti-MPIase antibody inhibits protein integration into inner membrane vesicles (INV) prepared from *E. coli*^7^, indicating that MPIase functions on the cytoplasmic side of inner membranes. Other than the accumulated lines of evidence of the involvement of MPIase *in vitro*, there is no evidence that MPIase is involved in protein integration *in vivo*, due to the lack of information on MPIase biosynthesis.

ECA (enterobacterial common antigen) is a glycolipid found in the outer membranes of Gram-negative bacteria^9^. Although ECA has the same trisaccharide unit as that of MPIase, knockout of each biosynthetic gene for ECA does not affect MPIase expression^7^, indicating the presence of an independent set of genes for MPIase biosynthesis. The structural differences between MPIase and ECA are the lengths of the glycan, and the linker between the glycan chain and the lipid moiety^7,9^. The numbers of repeating trisaccharide units in ECA are more heterogeneous and greater (n = 18~55), while that in MPIase is 9~11. The linker for ECA is monophosphate, while that for MPIase is pyrophosphate. This difference in the linker may reflect the differences in the biosynthetic pathways. Although the glycan unit for ECA is biosynthesized on a carrier lipid, undecaprenol^10^, depletion of 4-acetamido-4-deoxyfucose (Fuc4NAc) resulted in the accumulation of not only ManNAcA-GlcNAc-PP-undecaprenol but also ManNAcA-GlcNAc-PP-DAG (Fig. 1a); referred to as “DGP-disaccharide’^11^. This finding strongly suggests that ‘DGP-disaccharide’ is a possible intermediate for MPIase, and that MPIase is biosynthesized on phosphatidic acid, i.e., the first step for MPIase biosynthesis is generation of GlcNAc-PP-DAG (compound I, Fig. 1a). Therefore, we tried to identify the enzyme that catalyzes the generation of compound I, and found that CdsA, a CDP-DAG synthase involved in phospholipid biosynthesis^12,13^, is also involved in compound I biosynthesis. The combination of CdsA depletion and yeast Tam41p (a mitochondrial CDP-DAG synthase)^14^ expression revealed that MPIase is essential for membrane protein integration and therefore for cell growth. We also found that Cds1p, a eukaryotic homologue of CdsA^15,16^, has the ability to biosynthesize MPIase, suggesting that an MPIase homologue is conserved among eukaryotic cells.

## Results

### CdsA biosynthesizes compound I (GlcNAc-PP-DAG)

We assumed that the first pathway of MPIase biosynthesis is the generation of compound I. In the course of a search for biosynthetic substrates, we found that cytidine deprivation, with a cytidine auxotroph, JF618 (*pyrG51*)^17^, caused a reduction in the MPIase level detectable on immunoblotting with anti-MPIase antibody (Fig. 1b), suggesting that cytidinated compounds such as CDP-diacylglycerol (CDP-DAG) are utilized for MPIase biosynthesis. Under the cytidine deprivation conditions, accumulation of M13 procoat (precursor of the major coat protein of M13 bacteriophage) was also observed, strongly suggesting that MPIase is involved in protein integration *in vivo* (Fig. 1c). Upon membrane integration, M13 procoat undergoes processing of its signal (or leader) sequence, yielding mature M13 coat (Supplementary Fig. 1a). We assumed that compound I is generated through CDP-DAG, a precursor of all the phospholipids in *E. coli*^18^, of which biosynthesis is catalyzed by a CDP-DAG synthase, CdsA^13,19^. Upon CdsA overproduction, we observed a ~3-fold increase in both the CdsA and MPIase levels (Fig. 1d), suggesting that CdsA is involved in MPIase biosynthesis. YnbB is a paralog of CdsA (Supplementary Fig. 2). When YnbB was overexpressed, a similar increase in the MPIase level was also observed (Fig. 1d), indicating that YnbB possesses similar functions to CdsA.

An attempt to biosynthesize compound I *in vitro* by incubating CTP, PA and some possible GlcNAc donors [UDP-GlcNAc, CDP-GlcNAc and GlcNAc-1-phosphate (GlcNAc-P)] in the presence of INV and/or cytosol prepared from wild type *E. coli* cells was unsuccessful, presumably reflecting the very low level of MPIase expression^5^. On the other hand, when INV prepared from CdsA-overproducing cells were incubated with [^14^C]PA, CTP and a GlcNAc donor, we observed a compound similarly migrated on TLC to the synthetic reference of compound I (a brown spot) in a GlcNAc-P-dependent manner (Supplementary Fig. 3, Rf value = ~0.44 with Solvent system A). The compound was developed at the same position as the synthesized reference of compound I with two different kinds of solvent systems (see below). GN80 (*cdsA8*) is a pH-sensitive mutant of *cdsA* that accumulates PA under high pH conditions^20^. To monitor both compound I and CDP-DAG generation, we constructed RS80, a Cdh (CDP-DAG hydrolase)-deficient version of GN80. When INV prepared from RS80 were incubated with [^14^C]PA, CTP and GlcNAc-P, compound I was generated (Fig. 1e, lane 6, Rf value = ~0.24 with Solvent system B). The amount of compound I increased while that of CDP-DAG (Rf value = ~0.16) decreased, as the pH of the reaction mixture increased (Fig. 1e, lanes 4~6). The lower and upper products in lanes 4–6 migrated to the positions of synthetic references for CDP-DAG (lane 2) and compound I (lane 3), respectively. The identity of the generated compound I was further confirmed by co-development of the sample in lane 6 and the synthetic references (lanes 9 and 12), since the radioactive compound I and the synthetic reference for compound I showed a perfect match. Time course analysis revealed that compound I biosynthesis occurred mainly at high pH, while CDP-DAG was synthesized at pH 6.5 (Supplementary Fig. 4). The formation of compound I was dependent on CTP, GlcNAc-P, and INV (Supplementary Fig. 5). Immediately after the addition of GlcNAc-P during the biosynthesis reaction, compound I was generated (Supplementary Fig. 6). The structure of the compounds generated in the *in vitro* reactions involving the CdsA8 mutant were confirmed by LC-mass spectrometry (MS). Figure 1f shows a mass spectrum of the extract from the biosynthesis reaction mixture. The reaction mixture gave an [M-H]^−^ peak originated from compound I (*m/z* value of 930.5) at the retention time of 5.2 min, which is the same as that of the synthetic reference (Fig. 1f). The peak at 5.2 min included mainly two materials, of which the exact mass numbers were *m/z* 930.5131 and 952.5047, respectively. The former corresponded to compound I (C_43_H_82_NO_16_P_2_^−^, theoretical value 930.5114) and the latter corresponded to CDP-DAG (C_44_H_80_N_3_O_15_P_2_^−^, theoretical value 952.5070) (Supplementary Fig. 7). Moreover, MS/MS analysis from the former peak gave two fragment ions (*m/z* 709.4155 and 362.0046), which are identical with those from the synthetic reference (Supplementary Fig. 8). The peak of compound I was not observed when GlcNAc-P was omitted in the reaction mixture, while CDP-DAG was generated under the condition. (Supplementary Fig. 9). Partially purified CdsA8 was subjected to the reaction for compound I biosynthesis (Supplementary Fig. 10). Under these high pH conditions, the level of compound I increased with the reaction time, while that of CDP-DAG remained constantly low throughout the reaction time. The level of CDP-DAG (0.7~1.4 pmol/20 μL reaction) was similar to that of CdsA8 (0.8~1.6 pmol/20 μL reaction). These observations strongly suggest that CDP-DAG is synthesized and then converted to compound I on CdsA. They also suggest that CDP-DAG is not released from the CdsA8 mutant, unless it is converted to compound I. Consistent with the efficient generation of compound I at high pH by the CdsA8 mutant, we observed an increase in the MPIase level when GN80 was cultivated at a non-permissive pH (Fig. 1g). Sequencing of the *cdsA8* allele revealed that this mutation comprises an amino acid substitution of Tyr 207 to His, suggesting that deprotonation of the His residue at the mutation point impairs the CdsA function in CDP-DAG biosynthesis but not that in compound I biosynthesis.

### CdsA depletion causes MPIase depletion with accumulation of M13 procoat

**Figure 2:**
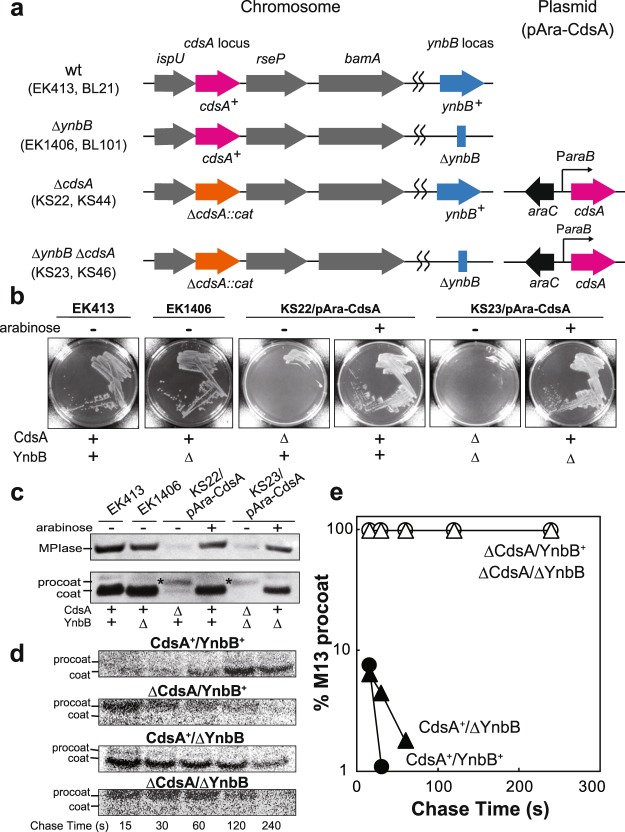
MPIase is essential for membrane protein integration *in vivo*. (**a**) Genetic organization of the *cdsA* and/or *ynbB* knockout mutants. In the Δ*cdsA* strains, CdsA is expressed from P*araB-cdsA* on plasmid pAra-CdsA. (**b**) Growth of the *cdsA* and/or *ynbB* knockout mutants. The indicated strains were streaked onto LB plates supplemented with 0.2% arabinose or not supplemented, and then incubated at 37 °C for 16 h. (**c**) MPIase depletion and M13 procoat accumulation upon CdsA depletion. Overnight cultures of the indicated strains harboring pMS119-PC were washed three times with fresh LB medium, followed by cultivation in the presence and absence of 0.2% arabinose at 37 °C. When the growth of the *cdsA* knockouts ceased, 1 mM IPTG was added to induce M13 procoat, and cultivation was continued for 1 h. Total cellular proteins were precipitated with TCA, followed by SDS-PAGE/immunoblotting using anti-MPIase (upper) and anti-M13 coat (lower) antisera. (**d**,**e**) M13 procoat is accumulated on MPIase depletion. Overnight cultures of KS22 and KS23 harboring pMS119-PC were washed three times with fresh M9 medium, followed by cultivation in the presence (CdsA^+^/YnbB^+^ and CdsA^+^/ΔYnbB) and absence (ΔCdsA/YnbB^+^ and ΔCdsA/ΔYnbB) of 0.2% arabinose, respectively. After 5-min induction with 1 mM IPTG, they were pulse-labeled for 30 s and then chased for the indicated periods. M13 procoat and coat were immunoprecipitated, followed by SDS-PAGE/autoradiography. The percentage of M13 procoat, obtained in (**d**), was determined and plotted against chase time (**e**).

We then constructed knockout mutants of *cdsA* and *ynbB* to determine whether or not CdsA and/or YnbB are involved in MPIase biosynthesis. The *cdsA* gene is the second one in the *ispU*-*cdsA*-*rseP*-*bamA* operon (Fig. 2a), all of them being essential, while *ynbB* is not essential^21^. In order not to disturb the gene expression of the operon, the open reading frame of *cdsA* was replaced with that of the *cat* gene (Supplementary Fig. 11) in the presence of the complementary plasmid pAra-CdsA, in which *cdsA* is placed under the control of the *ara* regulon (Fig. 2a). The *ynbB* knockout grew as well as the parent strain (Fig. 2b and Supplementary Fig. 12). On the other hand, both the *cdsA* knockout and the *cdsA/ynbB* double knockout were unable to grow in the absence of inducer arabinose (Fig. 2b and Supplementary Fig. 12), confirming that *cdsA* is essential. We found that MPIase was hardly detected in both the *cdsA* and *cdsA/ynbB* knockouts, when CdsA was depleted by cultivating them in the medium without arabinose (Fig. 2c, top). In the *ynbB* knockout, the MPIase level was not significantly changed (EK1406), indicating that the YnbB effect is marginal or auxiliary. These observations indicate that CdsA is a biosynthetic enzyme for MPIase. Next, we examined membrane integration of M13 procoat under the CdsA- and CdsA/YnbB-depleted conditions. When CdsA or CdsA/YnbB were depleted (i.e., when MPIase was depleted), accumulation of M13 procoat was observed, while M13 procoat was efficiently processed into the mature form in the presence of CdsA (Fig. 2c, bottom). Pulse-chase experiments revealed that upon MPIase depletion, M13 procoat was never processed into the mature form, but was quickly degraded (Fig. 2d,e). These observations strongly suggest that MPIase functions as a membrane protein integrase *in vivo*.

### MPIase is essential for cell growth and protein integration

**Figure 3:**
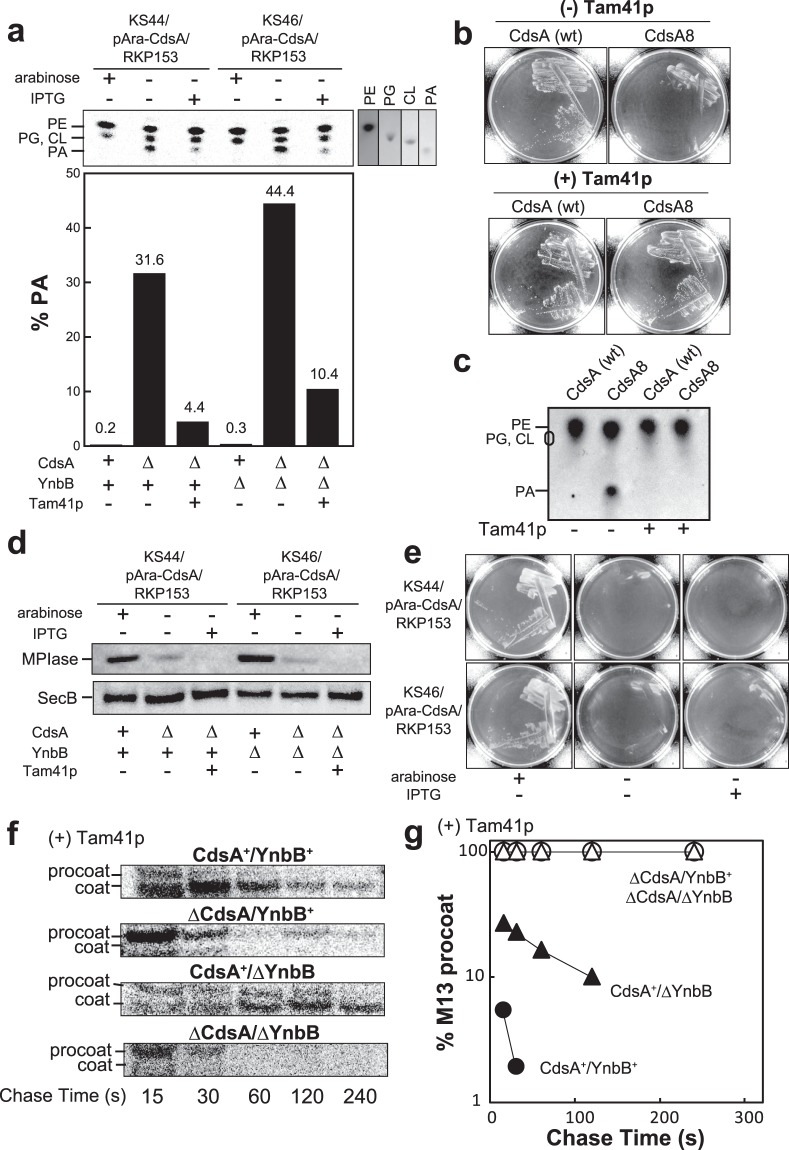
MPIase is essential for cell growth. (**a**) Accumulation of PA is relieved on Tam41p induction. The indicated cells harboring plasmids pAra-CdsA and RKP153 (for Tam41p expression from the T7 promoter), cultivated in LB medium in the presence and absence of 0.2% arabinose (for CdsA induction) or 0.1 mM IPTG (for Tam41p induction), were labeled with [^14^C] palmitic acid. Phospholipids were analyzed by TLC and autoradiography (top). Each reference was analyzed at the right. PE, phosphatidylethanolamine; PG, phosphatidylglycerol; CL, cardiolipin. The PA content in phospholipids was determined (bottom). (**b**) The pH-sensitive *cdsA8* mutation was complemented by Tam41p expression. EK23 (Δ*cdsA*) cells transformed with either pTet-CdsA (wt) or pTet-CdsA8 (CdsA8) with (right) or without (left) Tam41p expression (by pTac-Tam41p), were streaked onto LB plates at pH 8.5. (**c**) PA accumulation by *cdsA8* was relieved by Tam41p expression. Phospholipids in the indicated cells were analyzed by TLC and visualized with anisaldehyde/H_2_SO_4_. (**d**) Tam41p expression does not restore MPIase expression. The MPIase level in cells cultivated as in (**a**) was determined by immunoblotting. (**e**) MPIase is essential for cell growth. KS44 and KS46 harboring plasmids pAra-CdsA and RKP153 were streaked onto LB plates supplemented with 0.2% arabinose (for CdsA induction) or 0.1 mM IPTG (for Tam41p induction), and then incubated at 37 °C for 16 h. (**f**,**g**) M13 procoat is accumulated on MPIase depletion even in the presence of Tam41p. KS22 and KS23 cells harboring plasmids pAra-CdsA, pTet-Tam41p and pMS119-PC were subjected to pulse-chase experiments, as described in Fig. 2d. M13 procoat and coat were immunoprecipitated, followed by SDS-PAGE/autoradiography. The percentage of procoat, obtained in (**f**), was determined (**g**).

CdsA synthesizes CDP-DAG, a precursor of all phospholipids in *E. coli*^13,20,22^, which should be necessary for cell growth. To determine the essentiality of MPIase, we introduced mitochondrial Tam41p of yeast^23^ into the *cdsA*/*ynbB* knockout. Tam41p is a CDP-DAG synthase but exhibits no sequence homology to CdsA/YnbB^14^. When *TAM41* lacking the mitochondrial targeting signal^14^ was expressed in the *cdsA* or *cdsA/ynbB* knockout, PA accumulation caused by CdsA depletion was significantly relieved (Fig. 3a), indicating that Tam41p suppressed the defect in phospholipid biosynthesis. Functional expression of Tam41p in *E. coli* was also confirmed with the CdsA8 mutant. When CdsA8 was expressed in the *cdsA* knockout, no growth at high pH was observed (Fig. 3b). Under these conditions, severe accumulation of PA was observed (Fig. 3c). This growth defect and PA accumulation were clearly suppressed by Tam41p expression (Fig. 3b,c).

In the Δ*cdsA* strain, MPIase depletion was rather more complete upon Tam41p expression (Fig. 3d), indicating that Tam41p synthesizes CDP-DAG but not compound I. Under the MPIase-depleted conditions with Tam41p expression, the *cdsA* and *cdsA*/*ynbB* knockouts were unable to grow (Fig. 3e and Supplementary Fig. 13), indicating that MPIase is essential for cell growth.

M13 procoat accumulation was also observed under the MPIase-depleted conditions when Tam41p was expressed (Supplementary Fig. 14). Pulse-chase experiments again demonstrated that M13 procoat was never converted into the mature form but was quickly degraded upon MPIase depletion (Fig. 3f,g), indicating that MPIase is essential for M13 procoat integration *in vivo*. Unlike the cells without Tam41p expression (Fig. 2e), we observed procoat accumulation in the absence of YnbB (Fig. 3f,g), consistent with the finding that Tam41p overexpression renders MPIase depletion more complete (Fig. 3d).

### MPIase stimulates preprotein translocation *in vivo*

**Figure 4:**
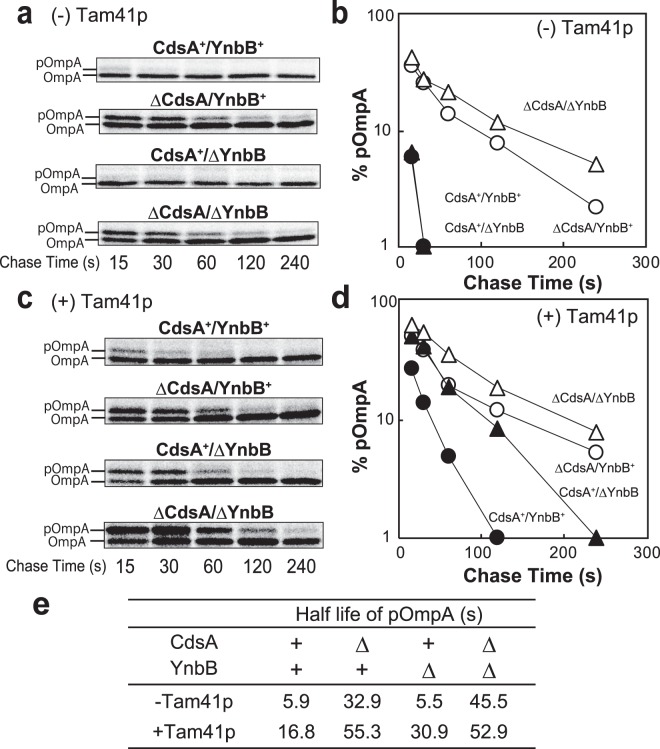
MPIase stimulates preprotein translocation. (**a**) pOmpA is accumulated on MPIase depletion. KS22 and KS23 cells were cultivated as described in the legend to Fig. 2d, except that IPTG induction was omitted. Cells were then pulse-labeled for 30 s, and then chased for the indicated periods. OmpA and pOmpA were immunoprecipitated, followed by SDS-PAGE/autoradiography. **(b)** The results in (**a**) were quantitated and plotted against chase time. **(c)** pOmpA is also accumulated on MPIase depletion in the presence of Tam41p. KS22 and KS23 cells harboring pTet-Tam41p were subjected to pulse-chase experiments as in (**a**). (**d**) The results in (**c**) were quantitated and plotted against chase time. (**e**) Summary of the pulse-chase experiments. The half-lives of pOmpA in (**b**,**d**) were calculated.

MPIase stimulates preprotein translocation^5,24^ by affecting the dimer structure of protein translocon SecYEG^24^. We examined the effect of MPIase depletion on preprotein translocation *in vivo* (Fig. 4). Processing of the signal (or leader) sequence of a presecretory protein, OmpA, was monitored as an index of preprotein translocation^25^ (Supplementary Fig. 1b) by means of pulse-chase experiments. We observed accumulation of pOmpA upon MPIase depletion, while most pOmpA materials were chased into mature OmpA in 4 min (Fig. 4a,b). Comparison of the half-life of pOmpA revealed that CdsA/YnbB depletion caused a ~10-fold decrease in the translocation rate (Fig. 4e), consistent with the results obtained in the *in vitro* reconstitution study^24^. Induction of Tam41p, which renders MPIase depletion more complete (Fig. 3d), caused more severe accumulation of pOmpA (Fig. 4c–e). Even in the absence of YnbB, pOmpA accumulation was observed, as seen for that of M13 procoat (Fig. 3f,g). These observations indicate that MPIase stimulates preprotein translocation, as revealed in the *in vitro* analysis^5,24^.

### INV prepared from MPIase-depleted cells are defective in protein integration and preprotein translocation

**Figure 5:**
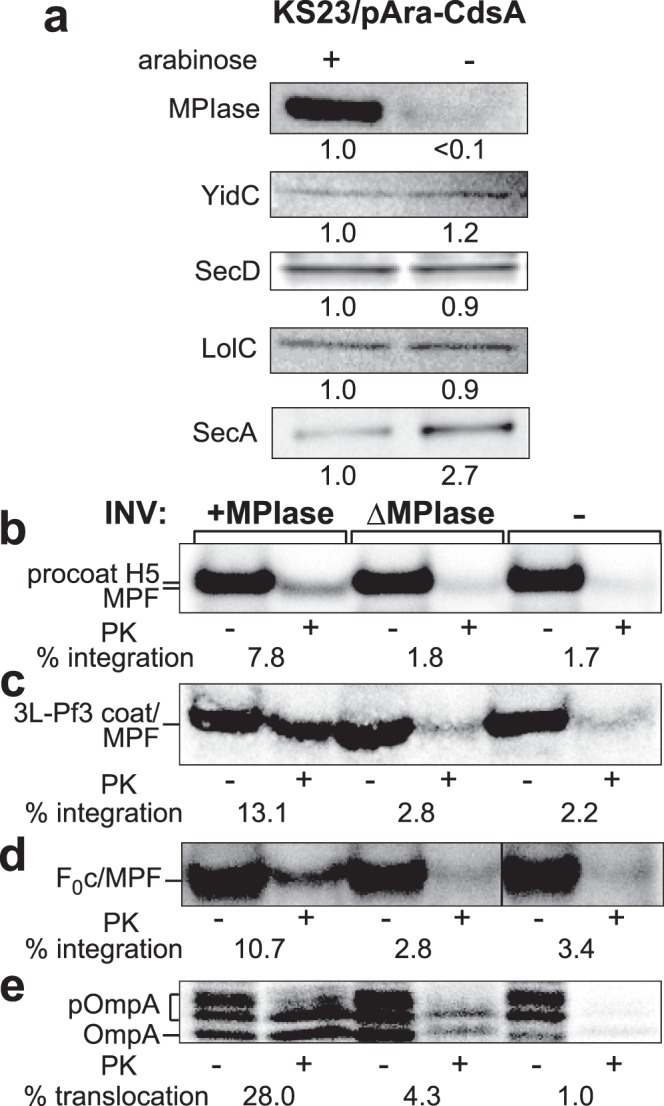
*In vitro* protein integration activity is impaired upon MPIase depletion. (**a**) The levels of some membrane proteins do not change upon MPIase depletion. INV were prepared from KS23 harboring plasmid pAra-CdsA cells cultivated in the presence or absence of 0.2% arabinose (*cdsA*^+^), followed by SDS-PAGE/immunoblotting using antisera against MPIase or the specified proteins. The relative amount as to the sample ‘+’ is indicated at the bottom of each gel. In the case of SecA, proteins from whole cells were applied. (**b**–**e**) Protein integration and translocation into ΔMPIase INV are impaired. Membrane proteins (M13 procoat (**b**), 3L-Pf3 coat (**c**), and F_0_c (**d**)), and presecretory protein pOmpA (**e**) were synthesized *in vitro* by means of the pure system in the presence of +MPIase and ΔMPIase INV. pOmpA gave several bands as structurally different forms^53,54^. After 30-min incubation at 37 °C, the reaction mixture was divided into two parts; one was analyzed as a translation control of 20% in the ‘−PK’ lanes, and the other was digested with proteinase K (PK) (+). At right, INV were not added (−). The integration and translocation activities are indicated at the bottom of each gel. In (**d**), a space for the duplicated sample (Supplementary Fig. 13) was removed.

Next, INV were prepared from MPIase-depleted cells, followed by *in vitro* integration assaying (Fig. 5). We observed efficient depletion of MPIase (Fig. 5a). Under the conditions for the MPIase depletion, the expression levels of some membrane proteins including YidC^26^, SecD^27^ and LolC^28^ remained unchanged (Fig. 5a). On the other hand, the SecA level increased upon MPIase depletion (Fig. 5a), consistent with the previous reports that SecA is induced when protein translocation is inhibited^29,30^. We then examined whether or not membrane integration of M13 procoat is inhibited *in vitro* upon MPIase depletion, as observed *in vivo*. To avoid complexity due to signal (or leader) processing, we used M13 procoat H5 with an uncleavable signal (or leader) sequence^31^. We observed a severe defect in M13 procoat H5 integration into MPIase-depleted INV, as reflected by the disappearance of the membrane-protected fragment (MPF)^5,32^ (Fig. 5b). The integration level for ΔMPIase INV was nearly the same as that in the absence of INV, indicating that M13 procoat integration is dependent on MPIase. These observations are consistent with the *in vivo* results (Figs 2 and 3). Essentially the same results were obtained when 3L-Pf3 coat (Fig. 5c) and the F_0_c subunit of F_0_F_1_-ATPase (Fig. 5d, Supplementary Fig. 15) were synthesized, confirming the previous findings on reconstitution^7,8,33^. Thus, that MPIase functions as a membrane protein integrase has been demonstrated both *in vitro* and *in vivo*. When pOmpA translocation was examined, we observed significant stimulation of pOmpA translocation in the presence of MPIase (Fig. 5e), again confirming the *in vivo* results (Fig. 4).

### Cds1p, a eukaryotic homolog of CdsA, can biosynthesize MPIase

**Figure 6:**
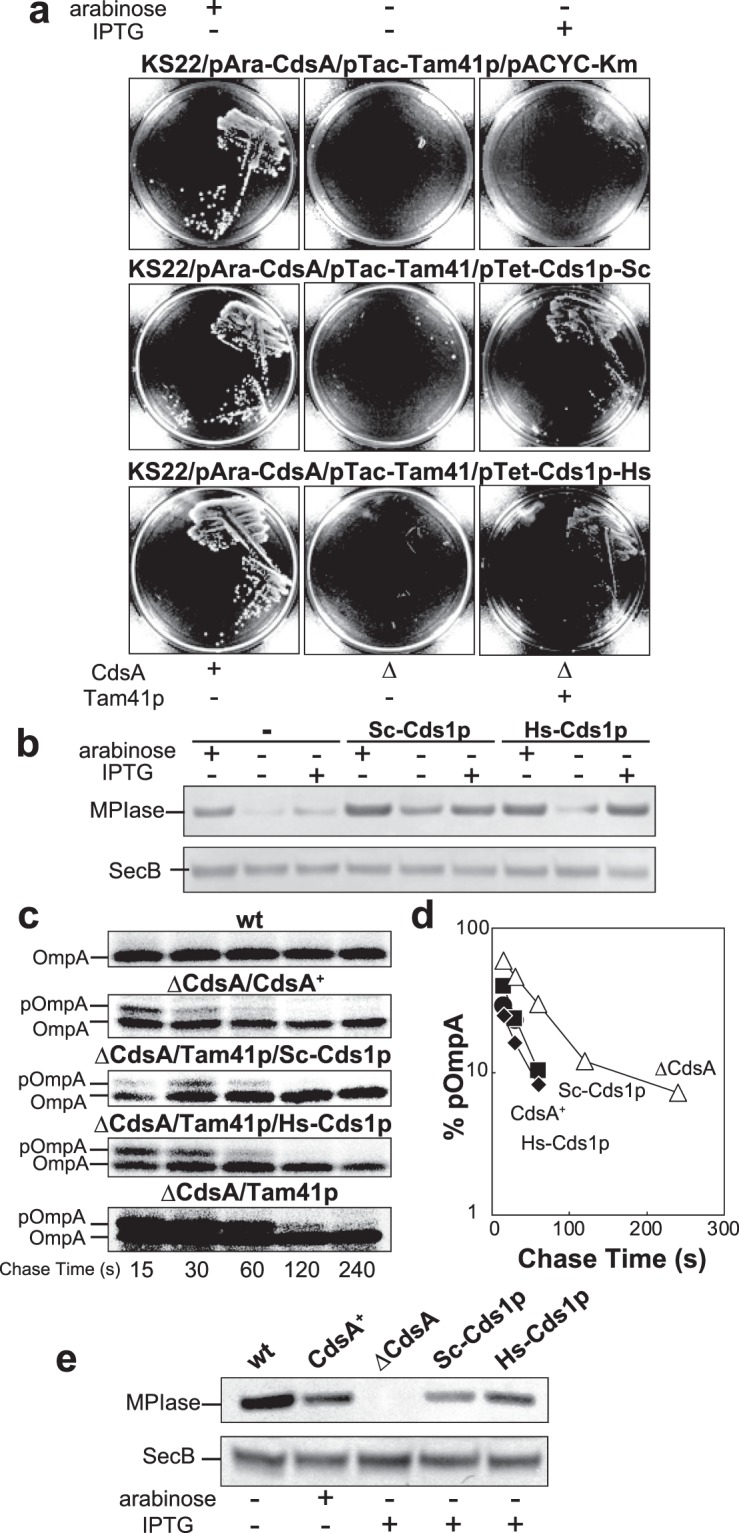
Eukaryotic Cds1p can synthesize MPIase. (**a**) Both Sc-Cds1p and Hs-Cds1p complement Δ*cdsA* when combined with Tam41p. KS22 cells harboring plasmids pAra-CdsA and pTac-Tam41p transformed with either pACYC-Km (top), pTet-Cds1p-Sc (middle), or pTet-Cds1p-Hs (bottom) were streaked onto LB plates supplemented with the specified inducer, followed by incubation at 37 °C for 16 h. (**b**) Both Sc-Cds1p and Hs-Cds1p restored the MPIase level when combined with Tam41p. The MPIase level in KS22 cells, in which the specified proteins had been induced, was determined by immunoblotting (top). The SecB level was also determined as a loading control (bottom). (**c**,**d**) pOmpA translocation was relieved on both Cds1p and Tam41p expression in the absence of CdsA. KS22 cells harboring plasmids pAra-CdsA and pTac-Tam41p plus either pACYC-Km, pTet-Cds1p-Sc, or pTet-Cds1p-Hs were pulse-labeled for 30 s and then chased for the indicated periods, as described in the legend to Fig. 4. pOmpA and OmpA were immunoprecipitated, followed by SDS-PAGE and autoradiography. EK413 (wt) cells were also analyzed as a CdsA^+^ control. The results in (**c**) were quantitated and plotted against chase time (**d**).

CdsA is ubiquitously conserved in all organisms. The eukaryotic homologue of CdsA is Cds1p (Supplementary Fig. 16). Next, we examined whether or not eukaryotic Cds1p can substitute for CdsA in the biosynthesis of MPIase. The *CDS1* gene of *S. cerevisiae* (*Sc-CDS1*) was introduced in the Δ*cdsA* knockout. Although Sc-Cds1p expression could not complement Δ*cdsA*, co-expression of Sc-Cds1p and Tam41p allowed the cell growth of the Δ*cdsA* knockout (Fig. 6a), suggesting that Sc-Cds1p biosynthesizes a sufficient amount of compound I but not of CDP-DAG. Immunoblot analysis confirmed that Sc-Cds1p synthesized MPIase when Tam41p reinforced the CDP-DAG synthase activity (Fig. 6b). Pulse-chase experiments (Fig. 6c,d) revealed that pOmpA processing was recovered to the control level (ΔCdsA/CdsA^+^) when both Sc-Cds1p and Tam41p were expressed in the Δ*cdsA* knockout, while only Tam41p expression did not suppress the translocation defect of pOmpA, indicating that Cds1p is responsible for the biosynthetic activity of MPIase. Under these conditions, the MPIase level induced by Sc-Cds1p and that by plasmid-encoded CdsA were similar, but these were lower than that in wt cells (Fig. 6e), explaining the partial recovery of pOmpA translocation (Fig. 6c). Essentially the same results were obtained using human Cds1p (Hs-Cds1p), i.e., Hs-Cds1p relieved the growth of the Δ*cdsA* knockout (Fig. 6a) and MPIase expression (Fig. 6b,e) together with Tam41p. Protein translocation (Fig. 6c,d) was also recovered on Hs-Cds1p expression together with Tam41p. Thus, a eukaryotic homolog Cds1p could be involved in MPIase biosynthesis.

## Discussion

In this paper, we have demonstrated that MPIase is essential for membrane protein integration *in vivo* and therefore for cell growth. Moreover, we have shown that MPIase stimulates preprotein translocation *in vivo*. This was achieved through the finding that CdsA is involved in MPIase biosynthesis, in addition to the well-defined function of a CDP-DAG synthase^20,22^, by which all the phospholipids in *E. coli* are biosynthesized^18^. The possibility that a defect in phospholipid biosynthesis caused by CdsA depletion perturbs membranes, which causes pleiotropic effects including the impairment of protein integration, could be excluded due to the result of introduction of Tam41p, which synthesizes CDP-DAG but not compound I. Moreover, the membrane integrity was maintained under our experimental conditions, irrespective of Tam41p induction, since pOmpA was finally processed into mature OmpA even under the MPIase depletion conditions (Fig. 4). Although MPIase-depleted cells should be lysed after prolonged cultivation, the levels of some membrane proteins remained unchanged under our experimental conditions (Fig. 5a). Although we could not rule out a malfunction of other integration factors that might indirectly cause a defect in protein integration, such effects would nevertheless be caused by MPIase depletion. Thus, all the findings that we have made by means of the *in vitro* reconstitution system as to MPIase^5–8,24^ were supported *in vivo*.

**Figure 7:**
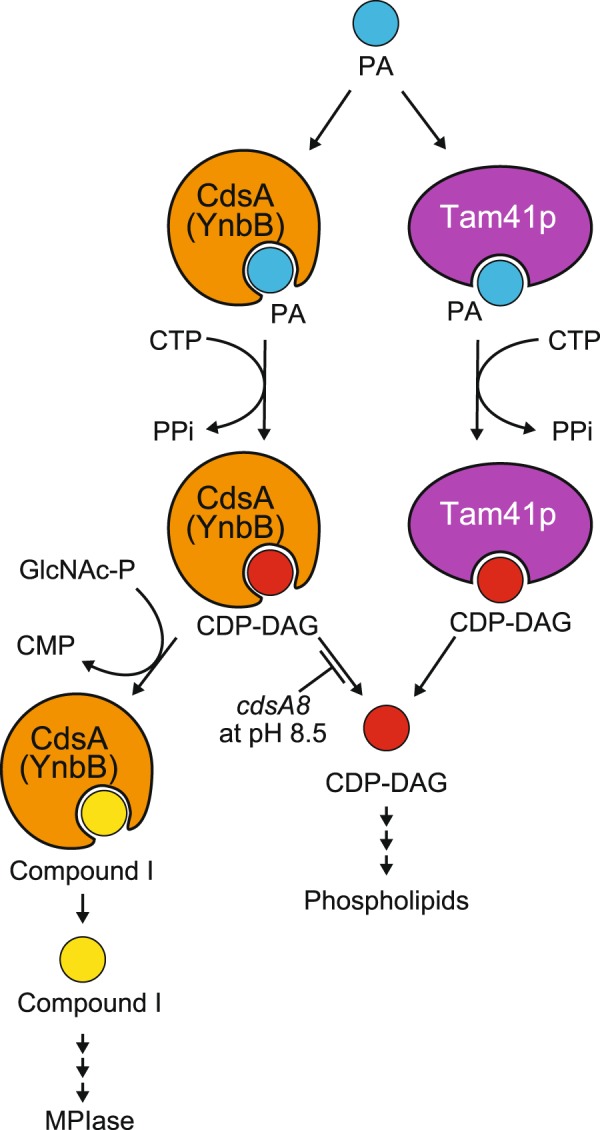
Working model for the CdsA/YnbB function with respect to MPIase biosynthesis. Both CdsA/YnbB and Tam41p biosynthesize CDP-DAG from CTP and PA. Unlike Tam41p, CdsA/YnbB also biosynthesize compound I immediately after CDP-DAG biosynthesis. The *cdsA8* mutation may retard the release of CDP-DAG from CdsA, which results in an increase in the efficiency of compound I biosynthesis.

We assumed that the initial step of MPIase biosynthesis is the formation of compound I, since a possible biosynthetic intermediate for MPIase, ManNAcA-GlcNAc-PP-DAG (‘DGP-disaccharide’), has been identified upon depletion of Fuc4NAc^11^. This compound was identified during the course of identification of a biosynthetic intermediate of ECA, of which the glycan is similar to that of MPIase^7^, but it turned out not to be an intermediate for ECA^11^. For ECA biosynthesis, the repeating unit of the glycan composed of three *N*-acetylated sugars is synthesized on a carrier lipid, undecaprenol, followed by polymerization of the glycan chain^9^. On the other hand, the identification of ManNAcA-GlcNAc-PP-DAG (‘DGP-disaccharide’) strongly suggests that the repeating unit for MPIase is synthesized on PA to allow its subcellular localization at the inside of inner membranes^7^, while the mechanism underlying glycan polymerization is totally unknown. Since the knockout mutant as to each gene for ECA biosynthesis still expressed MPIase at the wild-type level^7^, *E. coli* should possess a complete set of biosynthetic genes for MPIase. The *cdsA* gene is one of such MPIase-specific ones. We demonstrated that functionally expressed Tam41p suppressed the defect in phospholipid biosynthesis but not the defect in MPIase biosynthesis. These observations indicate that biosynthesis of CDP-DAG is not sufficient for compound I biosynthesis. On the other hand, the decrease in MPIase level in the *pyrG* mutant revealed that cytidine nucleotides are required for MPIase biosynthesis. Compound I biosynthesis, which was confirmed by MS analysis (Fig. 1f and Supplementary Figs 7 and 8), was dependent not only on GlcNAc-P but also on CTP in the *in vitro* reactions. Moreover, in the reactions involving an extract containing the pH-sensitive mutant CdsA8^20^, CDP-DAG was initially biosynthesized to the level of CdsA8, and subsequently compound I was generated, suggesting that CDP-DAG was converted into compound I through the incorporation of GlcNAc-P. The observation that nucleotide sugars such as UDP-GlcNAc and CDP-GlcNAc were not incorporated into PA or CDP-DAG yielding compound I (Supplementary Fig. 3) is consistent with the assumption that GlcNAc-P substitutes for the CMP moiety of CDP-DAG. MS analysis also demonstrated the generation of compound I in a GlcNAc-P-dependent manner (Supplementary Fig. 9). Considering that Tam41p could not biosynthesize compound I, this substitution should occur on CdsA (Fig. 7). The *cdsA8* mutant required co-expression of Tam41p for growth because CDP-DAG would hardly dissociate from CdsA, and a sufficient amount of CDP-DAG for the biosynthesis of bulk phospholipids was not provided. On the other hand, under the non-permissive conditions, MPIase was expressed at a higher level than in the wild-type, suggesting that low dissociation of CDP-DAG would be advantageous for GlcNAc-P incorporation (Fig. 7). The possibility cannot be excluded that some unknown factor(s) are necessary for GlcNAc-P incorporation. Nonetheless, the expression level of MPIase is quite low compared with that of bulk phospholipids; the MPIase level could be estimated to be <1/1,000 of that of phospholipids as a molar ratio^5–7^. MPIase has a labile pyrophosphate as a linkage between glycans and lipids^7^, while ECA has a monophosphate linkage^9^. This difference suggests that the presence of pyrophosphate could be involved in the control of MPIase level to keep it low, unlike ECA. The low level of MPIase expression is the possible reasons why we could not detect compound I formation under the wild-type conditions. In the *cdsA8* background, retardation of the release of CDP-DAG greatly helped to detect compound I. According to the crystal structure of CdsA^34^, the mutation point in the *cdsA8* allele (Tyr207His) lies in transmembrane stretch 6, which is close to the substrate-binding site. Under the non-permissive conditions at high pH, the His residue at the mutation point should be deprotonated, and therefore the mutation would stabilize the interaction between CdsA and CDP-DAG, thereby retarding the release of CDP-DAG (Fig. 7).

YnbB is a paralog of CdsA with a homologous region in the C-terminal half (Supplementary Fig. 2), while both YnbB and CdsA possess 8~9 transmembrane stretches. YnbB homologs are widely conserved among bacteria, although YnbB is not essential for growth. Since YnbB could not complement the Δ*cdsA* knockout even upon YnbB overproduction (data not shown), but its overproduction resulted in MPIase overproduction as well as CdsA overproduction, the YnbB function might be a backup for MPIase biosynthesis, but not for phospholipid biosynthesis. In this regard, the N-terminal half of CdsA might accelerate CDP-DAG biosynthesis of bulk phospholipids, while the role of the N-terminal half of YnbB is totally unknown. Similarly, Cds1p could biosynthesize a sufficient amount of MPIase but not of bulk phospholipids. Therefore, both YnbB and Cds1p have the ability to biosynthesize CDP-DAG and then compound I, however, the level of CDP-DAG biosynthesis is not sufficient for that of bulk phospholipids. In the presence of Tam41p expression, the effect of Δ*ynbB* was exaggerated; i.e., precursors of M13 coat and OmpA accumulated in the Δ*ynbB* and Tam41p^+^ background. These observations indicate that CdsA and YnbB compete with Tam41p for PA, resulting in a decrease in the MPIase level (Fig. 7). This competition is consistent with the finding that Tam41p overexpression renders MPIase depletion more complete (Fig. 3d).

MPIase co-precipitates with SecYEG, strongly suggesting their direct interaction^24^. Since YidC, which also interacts with SecYEG^35^, is involved in Sec-independent integration including M13 procoat integration^26,36^, it is likely that MPIase and YidC function in a concerted manner, suggesting that these factors directly interact. Recently, such collaboration between MPIase and YidC was clarified in the case of integration of F_0_c, the c subunit of F_0_F_1_ ATPase^8^. The MPIase function was clearly demonstrated in the *in vitro* analysis, which reflects an early stage of protein integration, since the appearance of MPF is used as an index of integration^5^. On the other hand, it is difficult to analyze the role of YidC in the *in vitro* system^5,33^, although YidC depletion causes a general defect in protein integration^26^. Based on the crystal structure of YidC, a detailed mechanism underlying YidC-dependent integration is proposed, where a hydrophilic cavity inside the membrane is important for integration of membrane proteins^37,38^. Taking these and the findings in the present study together, we propose that MPIase first drives an initial stage of integration, followed by transfer of membrane proteins to YidC to complete the integration reaction at a late stage. This model is consistent with the observation that upon YidC depletion M13 procoat is accumulated as an alkaline extraction-resistant form^36^. Under the conditions, M13 procoat integration proceeds with the MPIase function, but is not completed because of the lack of the YidC function, the precursor form remaining unprocessed.

Homologues of CdsA are ubiquitously conserved as Cds1p in eukaryotic cells^15,39^. If GlcNAc-P is sufficient for compound I synthesis without the requirement of additional factors, eukaryotic cells can synthesize compound I, since GlcNAc-P is also ubiquitously present. Although sugars in MPIase other than GlcNAc (Fuc4NAc and ManNAcA) seem specific in bacteria, and compound I might be an intermediate of a glycolipid with unknown functions, it is plausible that a compound homologous to MPIase, which is involved in membrane protein integration, is also ubiquitously conserved. Identification of such a homologue would shed new light on the molecular mechanisms underlying membrane protein integration in eukaryotic cells including those of higher plants and animals.

## Methods

### Materials

The *E. coli* strains (Table S1), plasmids (Table S2), and PCR primers (Table S3) used in this study are listed. SecA^40^ and SecB^41^ were purified as described. Antibodies against MPIase^7^, SecB^42^, SecD^27^, SecG^43^ and OmpA^44^ were raised in rabbits using the respective purified factors, while those against CdsA (Glu75~Ser92), M13 procoat (Ala24~Tyr44), LolC (Lys239~Glu255)^28^, and YidC (Lys382~Gln402) were raised against synthetic peptides corresponding to the specified regions. All the antibodies were obtained through the commercially available custom services. [Glycerol-^14^C(U)]-L-α-dipalmitoyl-phosphatidic acid (3.7~7.4 GBq/mmol), [^14^C(U)] palmitic acid (>18.5 GBq/mmol), and [^35^S] EXPRESS Protein Labeling Mix, a mixture containing both [^35^S] Met and [^35^S] Cys (~37 TBq/mmol), were obtained from Perkin Elmer, Inc. CDP-DAG, PA, PG and PE were from Avanti Polar Lipids, Inc. CL, GlcNAc-P, UDP-GlcNAc and L-arabinose were obtained from Sigma-Aldrich. CTP was from Roche Diagnostics. IPTG was from Wako Pure Chemical Industries, Ltd. CDP-GlcNAc (Supplementary Fig. 18) and the authentic reference of compound I (Supplementary Fig. 19) were chemically synthesized as described under Supplementary Methods. Protein A Sepharose was from GE Healthcare Life Sciences. TLC plates were from Merck.

### Biosynthetic formation of compound I

INV (2 mg/mL), prepared from RS80 (*cdsA8 Δcdh*), were solubilized with 1.5% octyl glucoside, and then mixed with [^14^C] PA (4 μM; ~23 kBq/mL), 1 mM CTP, 1 mM GlcNAc-P, 5 mM MgSO_4_ in 30 mM Bistris-H_2_SO_4_ (pH 6.5) or 30 mM Hepes-KOH (pH 7.5 or pH8.5), and 1.5% octyl glucoside. In some experiments, a cholate (6%) extract of INV, obtained by centrifugation (170,000 × g, 1 h, 4 °C), was used instead of INV. The reaction mixture (20 μL) was incubated at 37 °C. The reaction was terminated by adding 20 mM EDTA. An aliquot (2.5 μL) was analyzed by TLC using Solvent system B (chloroform/methanol/water/acetic acid: 5/5/0.89/0.11). The radioactive spots were visualized with a Phosphorimager (GE Healthcare Life Science), while a synthetic reference of compound I was stained with anisaldehyde-H_2_SO_4_ as described^5^.

### LC-MS analysis

INV (1 mg protein), prepared from RS80/pTac-CdsA8, were solubilized in 1.5% octyl glucoside, 10% glycerol, 1 mM dithiothreitol, 50 mM Tricine-KOH (pH 8.5). Solubilized membrane (0.5 mL), recovered by centrifugation (150,000 × g, 30 min, 4 °C), were mixed with dipalmitoyl-phosphatidic acid at 0.4 mg/mL, followed by dialysis against buffer C (50 mM Tricine-KOH (pH 8.5), 1 mM dithiothreitol) to form proteoliposomes with PA. Proteoliposomes were then recovered by centrifugation (150,000 × g, 60 min, 4 °C), suspended in buffer C at 5 mg protein/mL, and frozen-thawed-sonicated. Proteoliposomes (1.5 mg/mL) thus reconstituted were subjected to the reaction for compound I biosynthesis by incubating with 1 mM CTP, 1 mM GlcNAc-P, 1 mM ATP, 10 mM creatine phosphate, 10 μg/ml creatine kinase, 5 mM MgSO_2_, 30 mM K_2_SO_4_, 20 mM Tricine-KOH (pH 8.5) at 37 °C for 21 h. As a negative control, GlcNAc-P was omitted. After the reaction, proteoliposomes were recovered by centrifugation (150,000 × g, 60 min, 4 °C), and then suspended in MeOH (400 μL). The sonicated suspension was centrifuged (15,000 × g, 25 °C) for 1 min and supernatant (5 μL in each analysis) was subjected to the LC-MS analysis. High-resolution mass spectra of all products including compound I were obtained using an ion-trap time-of-flight mass spectrometer (Shimadzu LCMS-IT-TOF). A COSMOSIL 5C18-AR-II (2.0ID × 150 mm) column was used for separation of enzymatic products. The mobile phase 10 mM AcONH_4_ aq. (A) and acetone (B) were used for linear gradient elution (0-4-7-7.01-10 min, 60-100-100-60-60% (B)). Positive and negative ions were measured simultaneously. The parameters were as follows: nebulizer gas, 1.5 L/min; drying gas pressure, 190 kPa; CDL temperature, 200 °C; detector, 1.64 kV; and interface, 4.5 kV (positive mode) and −3.5 kV (negative mode).

### Construction of the Δ*ynbB* and Δ*cdsA* strains

Keio clone JW1406^21^ was used as a donor of the Δ*ynbB::kan* allele, which was transduced into EK413^43^ and BL21 (DE3)^45^ by means of the P1 phage, yielding EK4106 and BL101, respectively. The *kan* cassette in EK4106 and BL101 was removed using plasmid pCP20^46^, as described^47^, yielding KS21 (EK413 Δ*ynbB*) and KS42 (BL21 (DE3) Δ*ynbB*). The strategy for construction of the Δ*cdsA* knockout is illustrated in Supplementary Fig. 11. The Δ*cdsA::cat* allele was introduced by P1 transduction into EK413, KS21 (EK413 Δ*ynbB*), BL21 (DE3)^45^, and KS42 (BL21 (DE3) Δ*ynbB*), yielding KS22, KS23, KS44, and KS46, respectively. These strains harbored plasmid pAra-CdsA, and were constructed in the presence of 0.2% arabinose.

### Pulse-chase experiments

An overnight culture was washed with M9 medium three times to remove arabinose, followed by 1:1000 inoculation into the M9 medium supplemented with 18 amino acids (10 μM each) other than Met and Cys, and 0.01% yeast extract. Where specified, 0.2% arabinose was added to induce CdsA. When cell growth ceased upon CdsA depletion, [^35^S]-EXPRESS Protein Labeling Mix was added at ~100 kBq/ml to allow labeling for 30 s. Labeling was terminated by adding non-radioactive Met and Cys (12 mM each). At the specified chase time, 0.5 mL of the culture was withdrawn, and cellular proteins were precipitated with 5% TCA. The TCA precipitates were washed with acetone and ether successively, and then solubilized in 50 μL of 50 mM Tris-HCl (pH 7.5), 1% SDS, 1 mM EDTA. The samples were boiled for 3 min, and then diluted with 1 mL of 50 mM Tris-HCl (pH 7.5), 150 mM NaCl, 1% Triton X-100, 1 mM EDTA, 1 mM phenylmethylsulfonyl fluoride (PMSF). The insoluble materials were removed by centrifugation (10,000 × g, 5 min at 4 °C). Antiserum (2 μL) against either M13 coat or OmpA was added to the supernatant, and the mixture was incubated overnight at 4 °C. The mixture received 20 μL of protein A Sepharose (50% slurry), and then was kept at 4 °C for 60 min. After brief centrifugation, the resin was washed with 0.5 mL of 50 mM Tris-HCl (pH 7.5), 150 mM NaCl, 1% Triton X-100, and 0.5 mL of 50 mM Tris-HCl (pH 7.5), 1% Triton X-100, successively. The washed resin was suspended in 20 μL of the sample buffer used for SDS-PAGE and then boiled for 3 min. The immunoprecipitated materials were analyzed by SDS-PAGE and visualized with a Phosphorimager.

### Phospholipid analysis

An overnight culture, washed three times with LB medium, was inoculated into LB at the dilution of 1:500. When necessary, arabinose (0.2%) was added to induce CdsA. Tam41p was then induced with 0.1 mM IPTG for 30 min when cell growth ceased. Cells were labeled with [^14^C] palmitic acid (~50 kBq/mL) for 3 h at 37 °C. An aliquot (0.5 mL) of the culture was centrifuged to recover the cells. Phospholipids were then extracted, as described^48^. Phospholipids were analyzed by TLC using a solvent system (chloroform/methanol/water: 9/5/1) for development, followed by visualization using a Phosphorimager (GE Healthcare Life Science). Non-radioactive samples were visualized with anisaldehyde-H_2_SO_4_, as described^5^.

### Assaying of protein integration and translocation *in vitro*

Substrate membrane protein or presecretory protein was *in vitro* synthesized by means of a pure system, a reconstituted translation system composed of purified components^49^. The reaction mixture for the pure system (20 μL) containing INV (0.2 mg protein/mL), plasmids encoding the substrate protein under the T7 promoter, and [^35^S] methionine and cysteine (~10 MBq/mL) was incubated for 30 min at 37 °C. Cold methionine was omitted from the reaction. After the reaction had been terminated by chilling on ice, the mixture was divided into two parts. One part (3 μL) was treated with 5% trichloroacetic acid to precipitate proteins. The other part (15 μL) was mixed with an equal volume of proteinase K (1 mg/mL), followed by incubation for 20 min at 25 °C. After trichloroacetic acid (5%) had been mixed in, samples were incubated for 5 min at 56 °C to inactivate proteinase K, followed by recovery of proteins by centrifugation (10,000 × g, 5 min at 4 °C). Radioactive materials were separated by SDS-PAGE and then detected by autoradiography. Radioactivity was quantitated by means of ImageQuant software (GE).

### Construction of plasmids carrying genes for Cds1p

The CDS1 gene (1.3 kbp) of *S. cerevisiae* (*Sc-CDS1*) with a BamHI site at the 5′ end and a KpnI site at the 3′ end was PCR-amplified using a pair of primers (Sc-CDS1-5′ and Sc-CDS1-3′ comp). The gene fragment encoding *Sc-CDS1* was digested with BamHI, and then ligated to plasmid pUSI2 cut with BamHI and SmaI, yielding pTac-Cds1p-Sc. The gene fragment encoding Sc-Cds1p, obtained by digesting pTac-Cds1p-Sc with BamHI and SalI, was cloned into the same sites of pACYC-Km, yielding pTet-Cds1p-Sc. The human CDS1 gene (*Hs-CDS1*) was chemically synthesized, in which rare codons were replaced with major codons in *E. coli* (Supplementary Fig. 17). Plasmid pTet-Cds1p-Hs, which encodes *Hs-CDS1* under the control of the *tet* promoter, was constructed as described in the Supplementary Information.

### Other methods

SDS-PAGE was carried out using 12.5% acrylamide-0.27% *N*,*N*′-bismethyleneacrylamide containing 6 M urea for M13 (pro)coat, 3L-Pf3 coat and F_0_c, or not containing it for SecB, SecG and MPIase, as described^50^. Gels composed of 12.5% acrylamide-0.33% *N*,*N*′-bismethyleneacrylamide were also used^51^ to analyze other proteins. Bands on immunoblots were quantitated by means of a CS analyzer (ATTO). Proteins were quantitated using bovine serum albumin as a standard, as described^52^.

## Supplementary information

SUPPLEMENTARY INFO
