# Supplementary material for: CdsA is involved in biosynthesis of glycolipid MPIase essential for membrane protein integration in vivo
Source: Sci Rep. 2019 Feb 4;9:1372. doi: 10.1038/s41598-018-37809-8 (PMC6362211; doi:10.1038/s41598-018-37809-8)
Supplement: Supplementary file 1 — SUPPLEMENTARY INFO [file 41598_2018_37809_MOESM1_ESM.docx]

**Supplementary Tables, Figures, Methods, and References**

**CdsA is involved in biosynthesis of glycolipid MPIase essential for membrane protein integration *in vivo***

Katsuhiro Sawasato^1^, Ryo Sato^1^, Hanako Nishikawa^1^, Naoki Iimura^2^, Yuki Kamemoto^2^, Kohki Fujikawa^3^, Toshiyuki Yamaguchi^3^, Yutetsu Kuruma^4^, Yasushi Tamura^5^, Toshiya Endo^6^, Takuya Ueda^7^, Keiko Shimamoto^3^ & Ken-ichi Nishiyama^1,2,8,*^

^1^ The United Graduate School of Agricultural Sciences, Iwate University, Morioka, Iwate 020-8550, Japan.

^2^ Cryobiofrontier Research Center, Faculty of Agriculture, Iwate University, Morioka, Iwate 020-8550, Japan.

^3^ Bioorganic Research Institute, Suntory Foundation for Life Sciences, Seika-cho, Kyoto 619-0284, Japan.

^4^ Earth-Life Science Institute, Tokyo Institute of Technology, Meguro-ku, Tokyo 152-8550, Japan.

^5^ Faculty of Science, Yamagata University, Yamagata, Yamagata 990-8560, Japan.

^6^ Faculty of Life Sciences, Kyoto Sangyo University, Kita-ku, Kyoto 603-8555, Japan.

^7^ Department of Computational Biology and Medical Sciences Graduate School of Frontier Sciences, The University of Tokyo, Kashiwa, Chiba 277-8562, Japan

^8^ Department of Biological Chemistry and Food Science, Faculty of Agriculture, Iwate University, Morioka, Iwate 020-8550, Japan

*Correspondence should be addressed to K.N. (nishiyam@iwate-u.ac.jp).

**Supplementary Table 1. Strains used in this study.**

| Strains | Relevant genotype | Ref. |
| --- | --- | --- |
| JF618 | F^-^ *thr-1* *araC14* *leuB6*(Am) Δ(*gpt*-*proA*)62 *lacY1* *glnX44*(AS) *galK2*(Oc) λ^-^ *hisG4*(Oc) *cdd-6* *pyrG51* *rpsL31*(strR) *xylA5* *mtl-1* *pyrE60* *argE3*(Oc) *thiE1* | ^17^, CGSC |
| GN80 | F^-^ *thr-1* *araC14* *cdsA8* *lacY1* *tsx-78* Δ(*galK-attLAM*)*99* *eda-50* *hisG4* (Oc) *rfbC1* *rpsL136*(strR) *xylA5* *mtl-1* *thiE1* | ^20^, CGSC |
| RS80 | GN80 Δ*cdh* | This study |
| JM109 | *recA1*, *endA1*, *gyrA96*, *thi-1*, *hsdR17*(rK^-^ mK^+^), e14^-^ (*mcrA*^-^), *supE44*, *relA1*, Δ(*lac-proAB*)/F´ [*traD36*, *proAB*^+^, *lacI*^q^, *lacZ*ΔM15] | ^55^ |
| FS1576 | C600 *recD1009* | ^56^ |
| EK413 | MC4100 *ara^+^* | ^43^ |
| JW1406 | BW25113 Δ*ynbB::kan* | ^21^, Keio clone |
| EK1406 | EK413 Δ*ynbB::kan* | This study |
| KS21 | EK413 Δ*ynbB* | This study |
| KS22 | EK413 Δ*cdsA::cat* | This study |
| KS23 | EK413 Δ*cdsA::cat* Δ*ynbB* | This study |
| BL21 (DE3) | F^-^, *lon-11*, Δ(*ompT*-*nfrA*)885, Δ(*galM*-*ybhJ*)884, λDE3 [*lacI* *lacUV5-T7* *gene 1* *ind1* *sam7* *nin5*] Δ46 [*mal*^+^]K-12(λS) *hsdS10* | ^45^ |
| BL101 | BL21 (DE3) Δ*ynbB::kan* | This study |
| KS42 | BL21 (DE3) Δ*ynbB* | This study |
| KS44 | BL21 (DE3) Δ*cdsA::cat* | This study |
| KS46 | BL21 (DE3) Δ*cdsA::cat*, Δ*ynbB::kan* | This study |

**Supplementary Table 2. Plasmids used in this study.**

| Plasmid | Relevant description | Ref. |
| --- | --- | --- |
| pMS119-PC | Gene for M13 procoat is cloned under the control of the *tac* promoter, ampicillin resistant | ^57^ |
| pIVEX2.4b-Nde | Cloning vector containing the T7 promoter, ampicillin resistant | Roche |
| pET-15b | Cloning vector containing the T7 promoter, ampicillin resistant | Novagen |
| pT7-CdsA | *cdsA* was cloned into pET-15b under the control of the T7 promoter | This study |
| pT7-YnbB | *ynbB* was cloned into pET-15b under the control of the T7 promoter | This study |
| pTac-CdsA8 | *cdsA8* was cloned into pUSI2 under the control of *tac* promoter | This study |
| pCP20 | *flp* used to remove the *kan* cassette in the Keio clones, temperature-sensitive replicon | ^46^ |
| pUC19 | Cloning vector, ampicillin resistant | ^55^ |
| pUC19 (ΔNdeI-EcoRI) | Derivative of pUC19 with deletion between NdeI and EcoRI sites | This study |
| pCD1 | *cdsA* flanked by 2.5 kbp upstream and 2.2 kbp downstream region was cloned into pUC19 (ΔNdeI-EcoRI) | This study |
| pCD2 | Open reading flame of *cdsA* in pCD1 was replaced with that of *cat* | This study |
| pUSI2 | Cloning vector containing *tac* promoter, ampicillin resistant | ^58^ |
| pTac-CdsA | *cdsA* was cloned into pUSI2 under the control of *tac* promoter | This study |
| pKQ2 | Cloning vector containing arabinose regulon, ampicillin resistant | ^59^ |
| pKQ2-CdsA | *cdsA* was cloned into pKQ2 under the control of arabinose promoter | This study |
| pHP45Ω | Cloning vector, spectinomycin resistant | ^60^ |
| pAra-CdsA | *bla* on pKQ2-CdsA was replaced with *spc* | This study |
| RKP153 | *TAM41* without mitochondrial targeting signal is cloned under the control of the T7 promoter, ampicillin resistant | ^14^ |
| pACYC184 | Cloning vector, chloramphenicol resistant, tetracycline resistant | ^61^ |
| pUC4K | Cloning vector, kanamycin resistant | ^62^ |
| pACYC-Km | *cat* on pACYC184 was replaced with *kan* | This study |
| pTet-Tam41p | *TAM41* without mitochondrial targeting signal was cloned into pACYC-Km under the control of the *tet* promoter | This study |
| pTet-CdsA | *cdsA* was cloned into pACYC-Km under the control of the *tet* promoter | This study |
| pTet-CdsA8 | *cdsA8* was cloned into pACYC-Km under the control of the *tet* promoter | This study |
| pET3-H5 | Gene encoding M13 procoat H5^31^ is cloned under the control of the T7 promoter, ampicillin resistant | Prof. A. Kuhn |
| T7-7-3L-Pf3 | Gene encoding 3L-Pf3 coat^63^ is cloned under the control of the T7 promoter, ampicillin resistant | Prof. A. Kuhn |
| pIVEX-OmpA | *ompA* is cloned under the control of the T7 promoter, ampicillin resistant | ^64^ |
| pT7-UncE | *uncE* encoding F_0_c subunit of F_0_F_1_ ATPase is cloned under the control of the T7 promoter | ^8^ |
| pTac-Tam41p | *TAM41* without mitochondrial targeting signal was cloned into pUSI2 under the control of *tac* promoter | This study |
| pTac-Cds1p-Sc | *Sc-CDS1* was cloned into pUSI2 under the *tac* promoter | This study |
| pTet-Cds1p-Sc | *Sc-CDS1* was cloned into pUSI2 under the *tet* promoter | This study |
| pUC118 | Cloning vector, ampicillin resistant | ^65^ |
| pUC118-CDS1 (1-945) | DNA fragment corresponding to 1-945 bp of *Hs-CDS1* was cloned into pUC118 | This study |
| pUC118-CDS1 (946-1386) | DNA fragment corresponding to 946-1386 bp of *Hs-CDS1* was cloned into pUC118 | This study |
| pUC118-CDS1-Hs | *Hs-CDS1* was cloned into pUC118 | This study |
| pTac-Cds1p-Hs | *Hs-CDS1* was cloned into pUSI2 under the control of the *tac* promoter | This study |
| pTet-Cds1p-Hs | *Hs-CDS1* was cloned into pUSI2 under the control of the *tet* promoter | This study |

**Supplementary Table 3. Oligonucleotides used in this study.**

| Name | Sequence (5´->3´) | Purpose |
| --- | --- | --- |
| cdsA-5´ Nde | ctagtatgcatatgctgaagtatcgcctgat | Construction of pT7-CdsA |
| cdsA-3´ Sal | aaaagtcgacttaaagcgtcctgaataccagtaacaac | Construction of pT7-CdsA |
| ynbB-5´ Nde | ctagtatgcatatgctggaaaaatctctggc | Construction of pT7-YnbB |
| ynbB-3´ Sal | aaaagtcgacttagtaacagcagtagcgtataaaataaaaaa | Construction of pT7-YnbB |
| pKQ2 up comp | aaaaaagcttatacctacagcgtgagctatgagaaagcgc | Construction of pAra-CdsA |
| pKQ2 down | aaaagtcgactaggcgtatcacgaggccctttcgtcttca | Construction of pAra-CdsA |
| spc-5´ | aaaagtcgacgcttgtaaaccgttttgtgaaaaaattttt | Construction of pAra-CdsA |
| spc-3´ comp | tgattgagcaagctttatgcttgtaaaccgttttgtgaaa | Construction of pAra-CdsA |
| cdsA-5´ | ttttggatcctaggaggtttaaatttatgctgaagtatcgcctgatatctgcttttgtg | Construction of pKQ2-CdsA |
| cdsA-3´ comp | aaaagtcgacttaaagcgtcctgaataccagtaacaac | Construction of pKQ2-CdsA |
| 5.4 kbp-5´ | gccgccgctgacggaagaac | Construction of pCD1 |
| 5.4 kbp-3´ comp | tgcccggcaaggttgccgct | Construction of pCD1 |
| pCD1 up | aaaagcagatatcaggcgatacttcagcatatgcgacccccatcaggctg | Construction of pCD2 |
| pCD1 down | gttgttactagtattcaggggcgcctaacggaaggtaatatgctgagttt | Construction of pCD2 |
| cat-5´ | acttaagggttttctacatatggagaaaaaaatcactgg | Construction of pCD2 |
| cat-3´ comp | tgaagcttacgttggttcattaggcgccgccctgccactcatcgcagta | Construction of pCD2 |
| pACYC up comp | aaaaagatctgaaaatctcgataactc | Construction of pACYC-Km |
| pACYC down | aaaagaattcttttaaggcagttattggtgccc | Construction of pACYC-Km |
| kan-5´ | aaaagatctaggaggtttaaatttatgagccatattcaacgggaaac | Construction of pACYC-Km |
| kan-3´ comp | ggggaattcaaattagaaaaactcatcgagcatca | Construction of pACYC-Km |
| TAM41-5´ | aaaggatcctaggaggtttaaatttatgagaagttccatagatgatgctggcatta | Construction of pTac-Tam41p |
| TAM41-3´ comp | aaaaagtcgactttttagcttctcctcatcgattttagttttttggcccaag | Construction of pTac-Tam41p |
| Sc-CDS1-5´ | aaaaggatcctaggaggtttaaatttatgtctgacaaccctgagatgaaaccacatgg | Construction of pTet-Cds1p |
| Sc-CDS1-3´ comp | aaaaagatctggtaccttttcaagagtgattggtcaatgatttcttggtcaca | Construction of pTet-Cds1p |


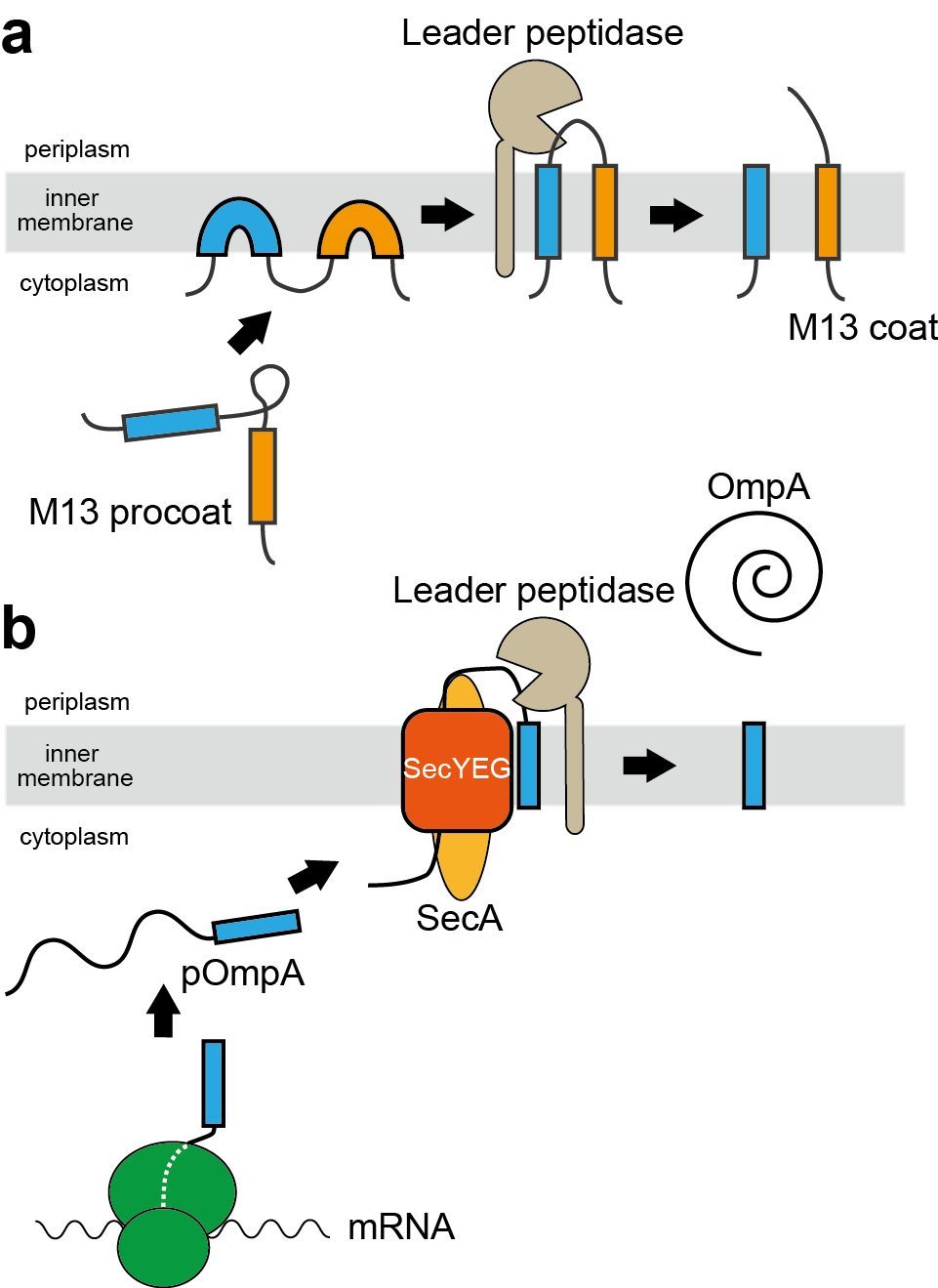


**Supplementary Fig. 1. Signal (or leader) processing upon membrane integration (a) and translocation (b).** Membrane proteins (such as M13 procoat in (a)) and presecretory proteins (such as pOmpA in (b)) with signal (or leader) peptide are processed to mature proteins upon membrane insertion into (a) and translocation across (b) the inner membrane, respectively. Since the catalytic domain of leader peptidase is localized at the periplasmic surface of the inner membrane, the cleavage of the signal (or leader) peptide represents protein integration or translocation. Signal (or leader) peptide is shown as blue boxes.

**
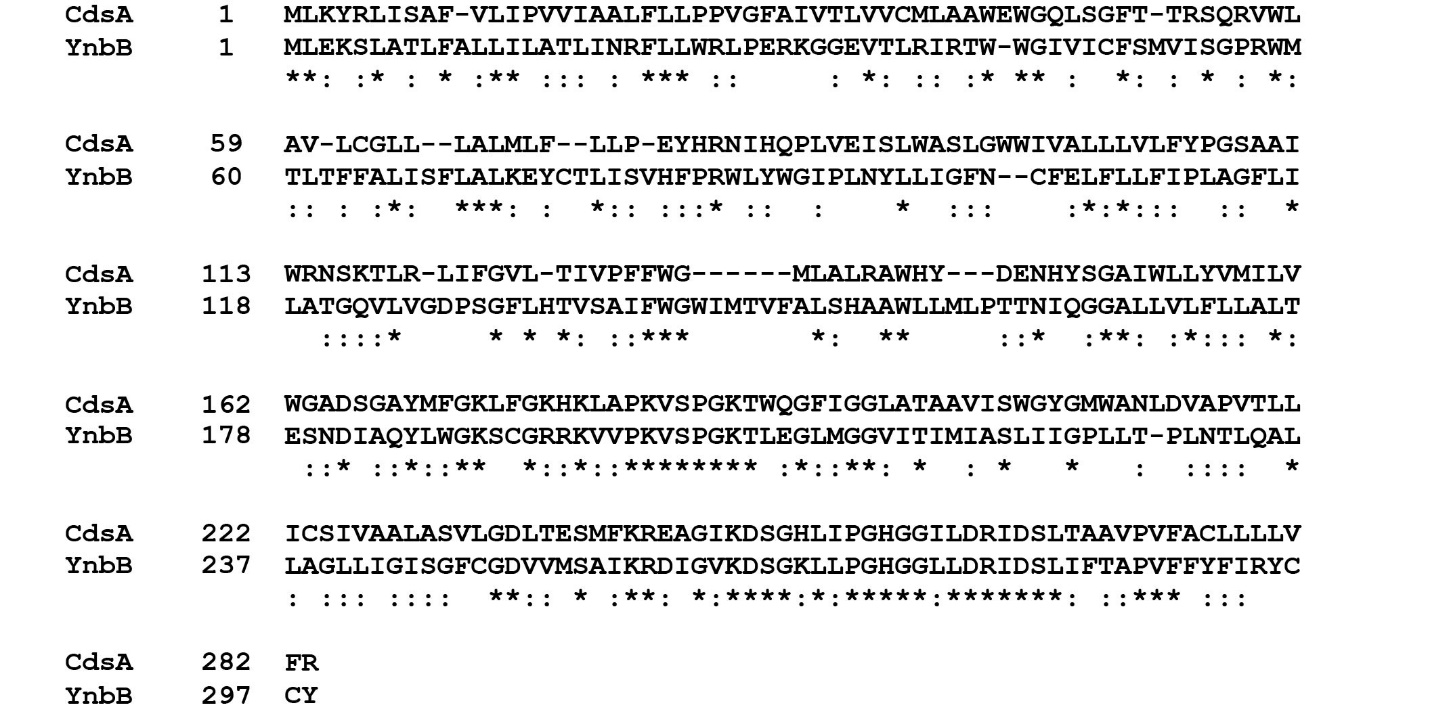
**

**Supplementary Fig. 2. YnbB is a CdsA homologue.** Amino acid alignment for *E. coli* CdsA and YnbB is shown. Asterisks and colons represent identical and similar amino acids, respectively. Identity: 31.5% (192/279); Similarity: 68.8% (192/279).


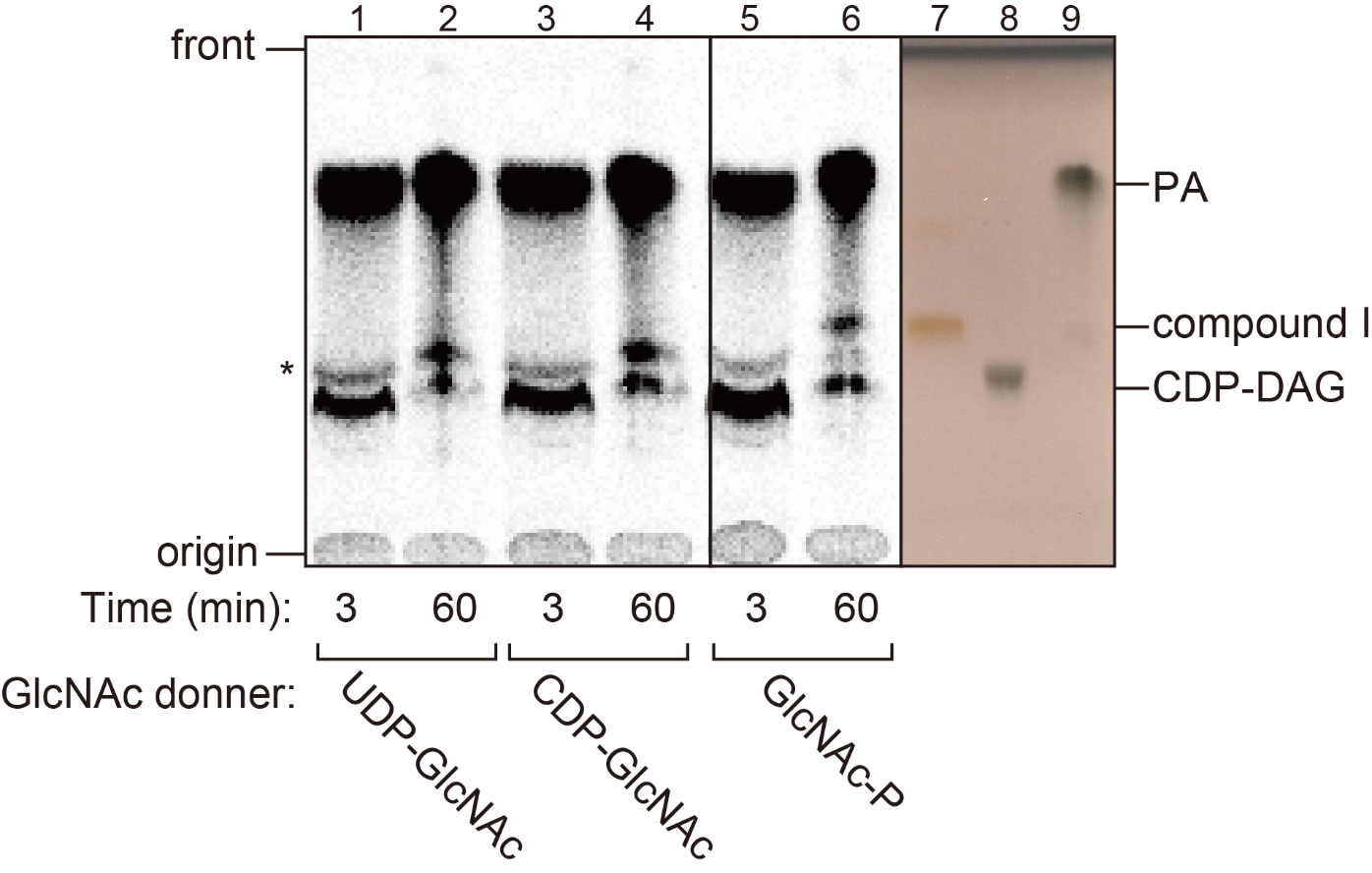


**Supplementary Fig. 3. GlcNAc is a donor for compound I biosynthesis.** INV (2 mg/mL) and cytosol (5 mg/mL) prepared from JM109/pTac-CdsA were mixed with [^14^C] PA (4 μM; ~23 kBq/mL), 2 mM CTP in 5 mM MgSO_4_, 90 mM KCl, 1.5% octyl glucoside, 30 mM Hepes-KOH (pH 7.5) in the presence of the specified GlcNAc donor at 2 mM. The reaction mixture (20 μL) was incubated at 37°C. At the indicated period, an aliquot (5 μL) was withdrawn and spotted on the TLC plates. TLC was then developed in three kinds of solvents (chloroform/methanol: 4/1, chloroform/methanol/water: 10/5/1, chloroform/methanol/ acetic acid: 10/5/1), successively (Solvent system A). The radioactive materials were visualized by autoradiography. The synthesized references (compound I, lane 7; CDP-DAG, lane 8; PA, lane 9) were also analyzed in a same way, and stained by anisaldehyde-H_2_SO_4_. The positions of origin, front, PA, CDP-DAG, and compound I are indicated. Asterisk, a degradation product from [^14^C] PA. Note that CDP-DAG generated in each reaction was readily hydrolyzed by Cdh, a CDP-DAG hydrolase.


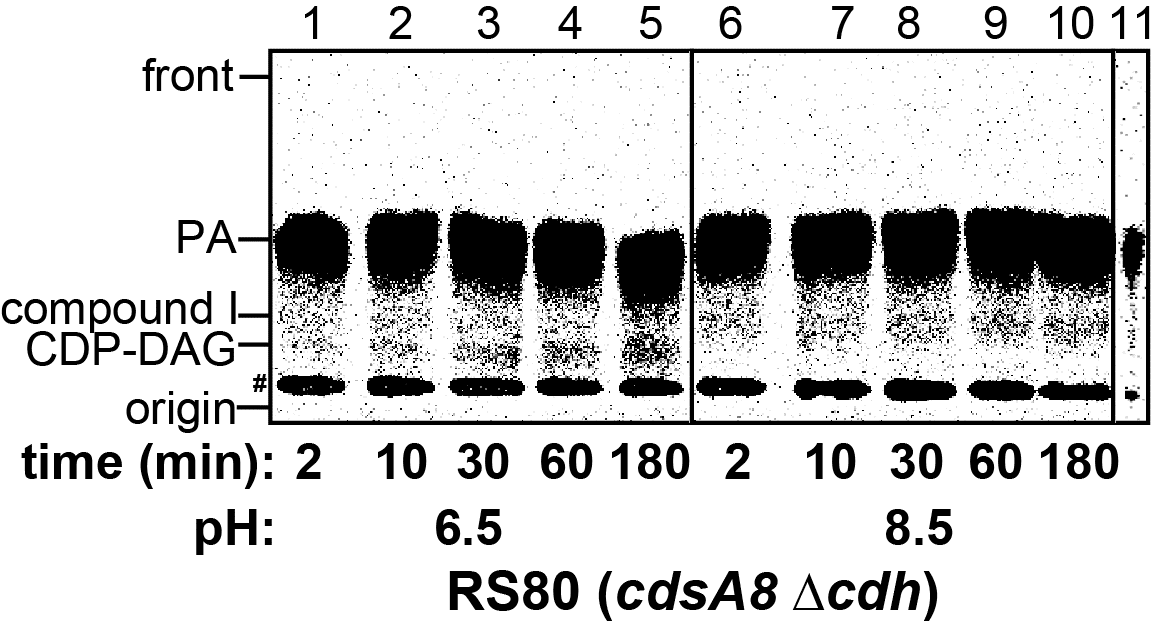


**Supplementary Fig. 4. Time course analysis of CDP-DAG/compound I biosynthesis.** INV prepared from RS80 (*cdsA8* Δ*cdh*) were mixed with [^14^C] PA, CTP (1 mM), GlcNAc-P (1 mM) as described in the legend to Supplementary Fig. 3. The reaction mixture (30 μL) was incubated at 37°C either at pH 6.5 (left) or 8.5 (right). At the indicated period, an aliquot (2.5 μL) was withdrawn and spotted on the TLC plates. TLC was then developed with Solvent system B (chloroform/acetone/80% acetonitrile: 1/6/6). The radioactive materials were visualized by autoradiography. In lane 11, [^14^C] PA was analyzed. The positions of origin, front, PA, CDP-DAG, and compound I are indicated. The contaminants in [^14^C]PA are indicated by ‘#’.

**Supplementary Fig. 5. Compound I biosynthesis is dependent on CTP, GlcNAc-P and INV.** INV prepared from GN80 (*cdsA8*)/pTac-CdsA8 were incubated with [^14^C] PA, CTP and GlcNAc-P under the specified conditions, as described in the legend to Supplementary Fig. 3. TLC was developed with Solvent system B (chloroform/acetone/80% acetonitrile: 1/6/6). Positions of compound I and CDP-DAG are indicated by an arrow. In lane 1, [^14^C] PA was analyzed. Degradation product from [^14^C] PA by an asterisk.

**Supplementary Fig. 6. GlcNAc-P-dependent conversion of CDP-DAG into compound I.** Reaction mixture without GlcNAc-P was initiated at -60 min. At 0 min, GlcNAc-P was added. Lane 1, [^14^C]PA alone. The positions of the products are indicated.


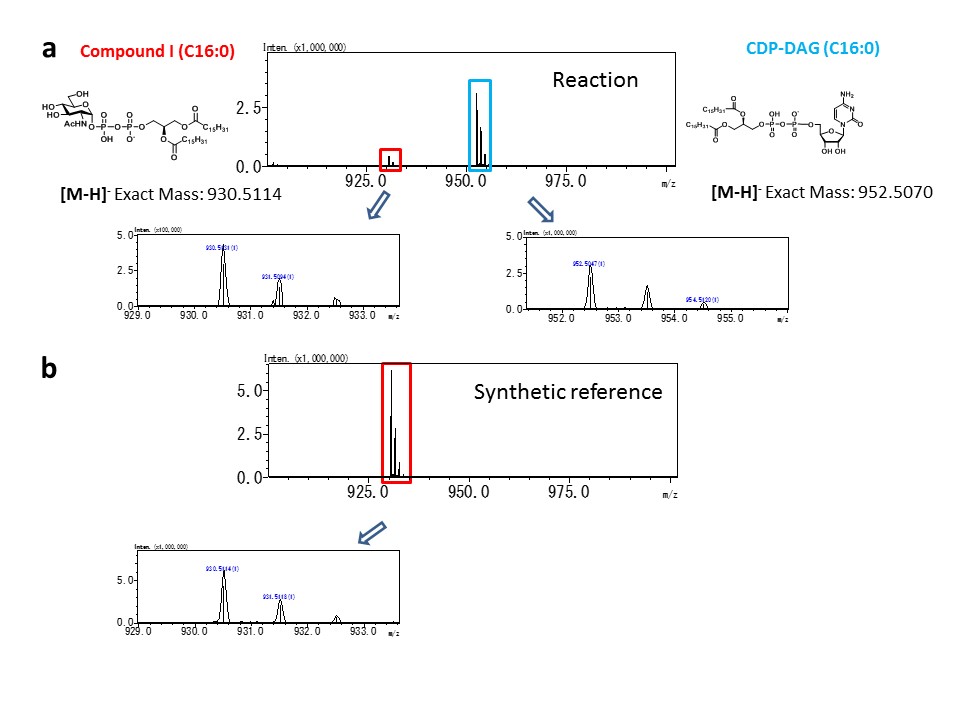


**Supplementary Fig. 7. Detection of compound I and CDP-DAG on LC-MS analysis. (a)** Mass spectrum from the peak at 5.2 min of HPLC of the biosynthetic reaction mixture and the magnified views at *m/z* 930 and *m/z* 952, corresponding to Compound I and CDP-DAG, respectively. **(b)** Mass spectrum from the peak at 5.2 min of HPLC of the synthetic reference and the magnified views at *m/z* 930.

**
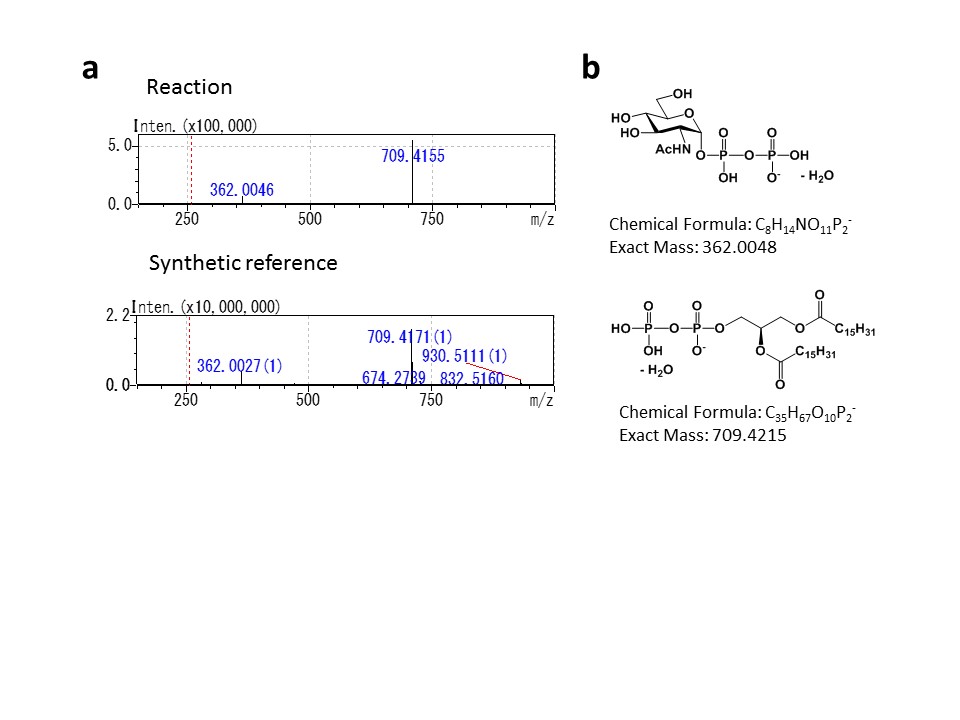
**

**Supplementary Fig. 8. MS/MS analysis of compound I.** **(a)** The MS/MS spectrum from *m/z* 930.5 in the negative mode. (upper) the biosynthetic reaction mixture. (bottom) the synthetic reference. **(b)** The putative structure of fragment ions.


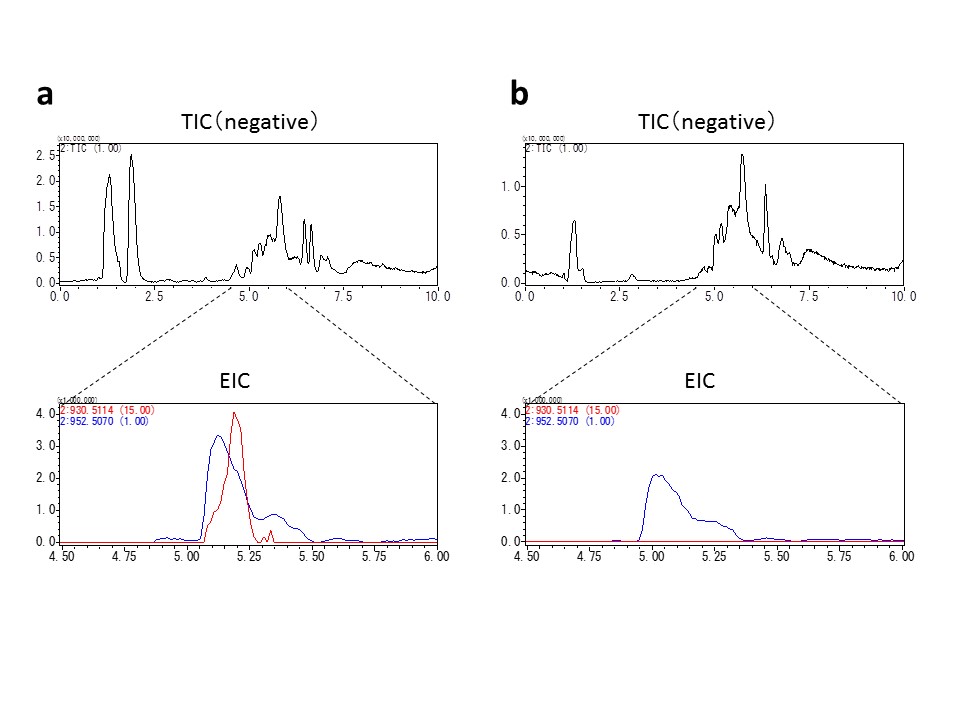


**Supplementary Fig. 9. GlcNAc-P-dependent formation of compound I. (a)** The ion chromatogram of the biosynthetic reaction mixture with GlcNAc-P. (upper) TIC (negative mode) (bottom) The magnified view of EIC (red: *m/z* 930.5114 and blue: *m/z* 952.5070). **(b)** The ion chromatograms of the biosynthetic reaction mixture without GlcNAc-P.

**Supplementary Fig. 10. Partially purified GlcNAc-P and INV.** Time course analysis using a cholate (6%) extract of INV from RS80/pTac-CdsA8. Compound I was biosynthesized at pH 8.5 as in Fig. 1e. Lane 1, [^14^C]PA alone. The positions of the products are indicated.


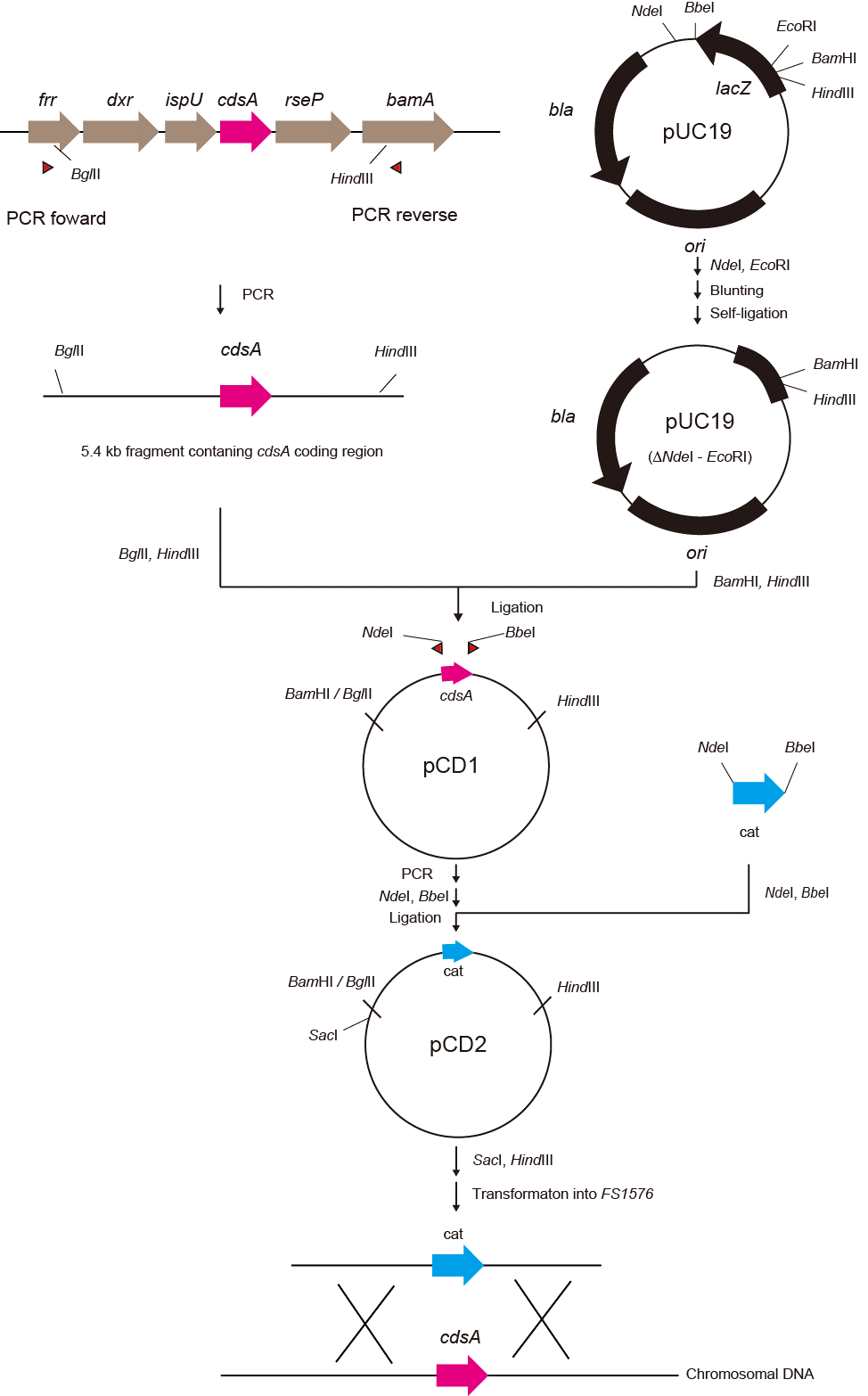


**Supplementary Fig. 11. Construction of the *cdsA* knockout.** Scheme to construct the Δ*cdsA::cat* mutant is shown. Details are shown in the Methods section.


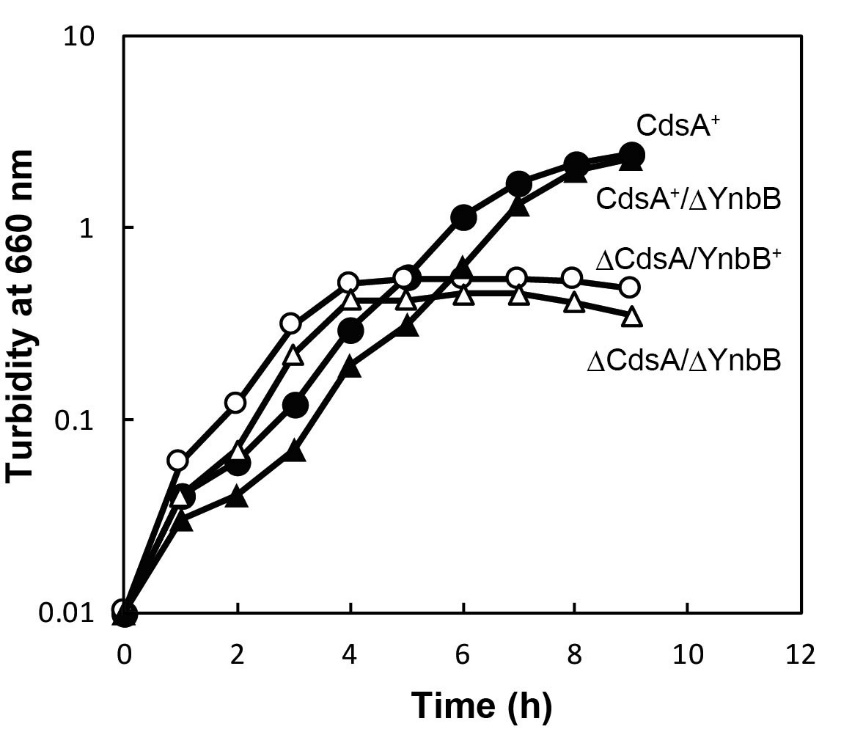


**Supplementary Fig. 12. CdsA is essential for cell growth but YnbB is not in liquid media.** Overnight culture of KS22(Δ*cdsA::cat*)/pAra-CdsA (for CdsA^+^/YnbB^+^ and ΔCdsA/YnbB^+^) and KS23(Δ*cdsA::cat* Δ*ynbB*)/pAra-CdsA (for CdsA^+^/ΔYnbB and ΔCdsA/ΔYnbB) grown in LB medium supplemented with 0.2% arabinose were washed three times with fresh LB medium, followed by inoculation to LB medium or LB medium supplemented with 0.2% arabinose. The growth of cells for specified conditions were monitored.


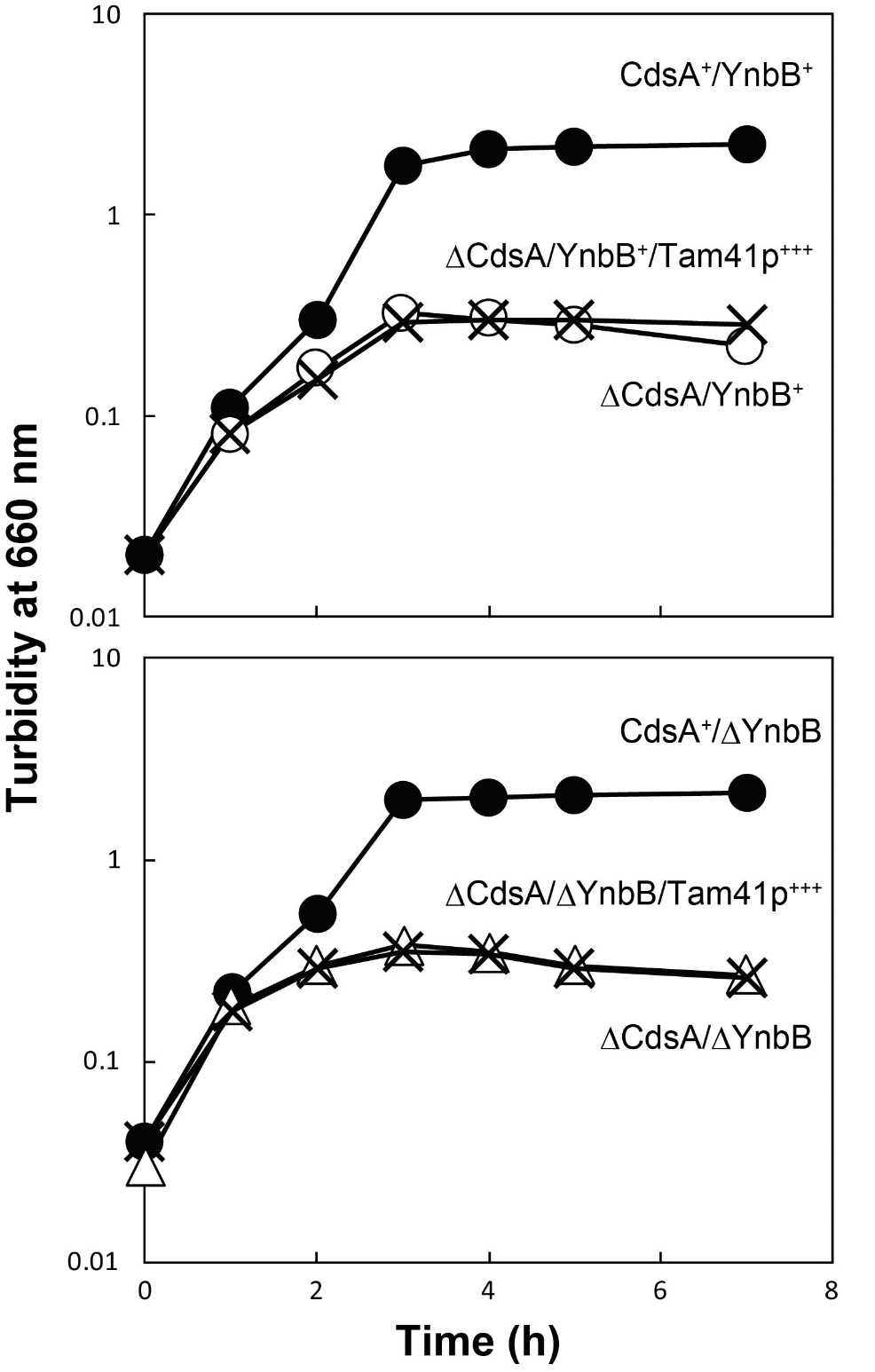


**Supplementary Fig. 13. Tam41p does not restore the growth of the Δ*cdsA* knockout in liquid media.** Overnight culture of KS44 (Δ*cdsA::cat*)/pAra-CdsA and RKP153 (for CdsA^+^/YnbB^+^, ΔCdsA/YnbB^+^ and ΔCdsA/YnbB^+^/Tam41p^+++^) (upper graph) and KS46(Δ*cdsA::cat* Δ*ynbB*)/pAra-CdsA and RKP153 (for CdsA^+^/ΔYnbB, ΔCdsA/ΔYnbB and ΔCdsA/ΔYnbB/Tam41p^+++^) (lower graph) grown in LB medium supplemented with 0.2% arabinose were washed three times with fresh LB medium, followed by inoculation to LB medium, LB medium supplemented with 0.2% arabinose (for CdsA induction) or LB medium supplemented with 1 mM IPTG (for Tam41p induction). The growth of cells for specified conditions were monitored.


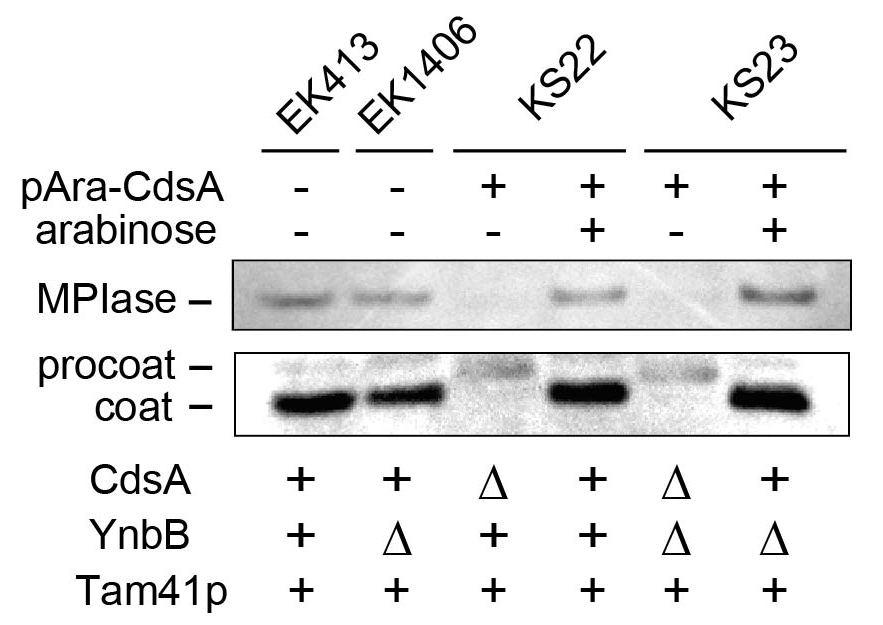


**Supplementary Fig. 14. M13 procoat accumulation upon MPIase depletion with Tam41p expression.** Indicated cells harboring pMS119-PC and pTet-Tam41p were used. EK413 and Ek4106 were cultivated in LB medium. KS22 and KS23 grown in the presence of 0.2% arabinose were washed three times with fresh LB medium, followed by inoculation in LB medium supplemented with 0.2% arabinose or not supplemented as indicated. M13 procoat was then induced with 1 mM IPTG for 1 h. The MPIase level (upper panel) and M13 (pro)coat (lower panel) were analyzed by immunoblotting.

**INV: +MPIase ΔMPIase ΔMPIase -**

**F_0_c/MPF**

**PK: - + - + - + - +**

**% integration: 10.7 2.8 3.5 3.4**

**Supplementary Fig. 15. Original image for Fig. 5d.** The autoradiograph of the integration assay for F_0_c is shown. Integration into ΔMPIase INV was duplicated. The space surrounded by white boxes are shown in Fig. 5d.


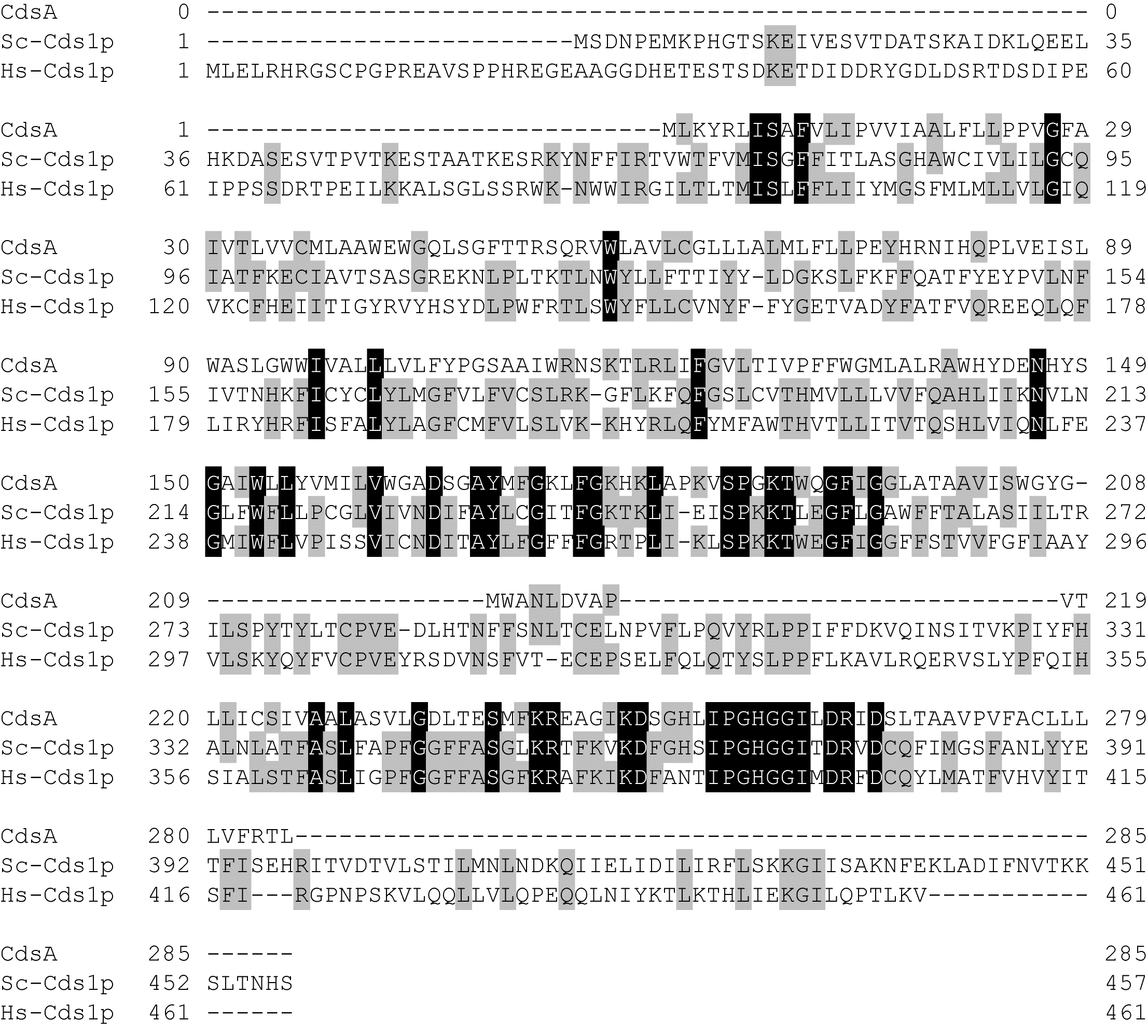


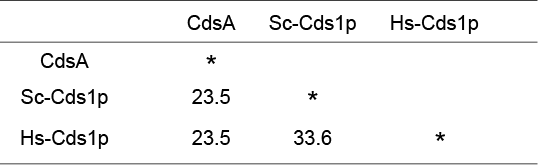


**Supplementary Fig. 16. Cds1p is a eukaryotic homologue of CdsA.** Alignment of amino acid sequence of *E. coli* CdsA, yeast Cds1p (Sc-Cds1p) and human Cds1p (Hs-Cds1p) is shown. Identical amino acids among three proteins (black) and two proteins (gray) are indicated. Percentages of identity between two of three proteins were summarized at bottom.

*Hs-CDS1* (1-945 bp)

aaaaggatcc aggaggttta aattt*atg*tt ggagctgcgc caccgtggca gctgcccggg

cccgcgcgaa gcggtgtcgc cgccgcaccg cgagggcgag gcggccggcg gcgaccacga

aaccgagagc accagcgaca aagaaaccga tattgatgac cgctatggcg atttggatag

ccgtaccgat agcgatattc cggaaattcc gccgagctca gatcgcaccc gtgagattct

caaaaaagct ctgagcggtt taagctcacg ttggaaaaac tggtggattc gtggcattct

caccctgacc atgatctcgt tgtttttcct gatcatctat atgggcagct tcatgctgat

gcttcttgtt ctgggcatcc aagtgaaatg cttccatgaa attatcacca ttggttatag

agtctatcat agctatgatc tgccgtggtt tcgcaccctg agttggtact ttctgttgtg

tgtaaactac tttttctatg gcgagaccgt agctgattat tttgctacct ttgttcaacg

cgaagaacaa cttcagttcc tcattcgcta ccatcgtttt atatcatttg ccctctatct

ggcaggtttc tgcatgtttg tactgagttt ggtgaagaaa cattatcgtc tgcagtttta

tatgttcgca tggacccatg tcaccttact gattaccgtc acccagtcac accttgtcat

ccaaaatctg tttgaaggca tgatttggtt ccttgttccg atttcaagtg ttatctgcaa

tgacattacc gcttaccttt ttggcttttt ttttggccgc actccgttaa ttaagttgag

cccgaaaaag acctgggaag gcttcattgg tggtttcttt agcaccgttg tgtttggctt

cattgctgcc tatgtgttaa gcaaatacca gtactttgtc tgcccggtgg aataccgtag

tgatgttaac ggtaccaaaa

*Hs-CDS1* (946 - 1386 bp)

aaaaggatcc gttaacagct tcgtgaccga atgtgagccg tcagaacttt tccagcttca

gacctactca cttccgccgt ttctgaaggc agtcttgcgc caggaacgtg tgagcttgta

cccgttccag atccacagca ttgcactgtc aacctttgca agcttaattg gcccgtttgg

cggcttcttt gctagtggct tcaaacgcgc cttcaaaatc aaggattttg caaataccat

tccgggccat ggtgggatta tggaccgttt tgattgtcag tatttgatgg caacctttgt

acatgtgtac atcaccagtt ttattcgcgg cccgaatccg agcaaagtgc tacagcagtt

gttggtgctt caaccggaac agcagttaaa tatatataaa accctgaaga cccatctcat

tgagaaagga atcctacaac cgaccttgaa ggta*taa*aga tctggtacca aa

**Supplementary Fig. 17. DNA Sequence of chemically synthesized gene encoding Cds1p Hs.** Nucleotide sequence encoding *Hs-CDS1* (1-945 bp) (upper) and *Hs-CDS1* (946-1386 bp) (lower) is shown. Attached restriction sites are underlined, while initiation and stop codons are italicized.

**Supplementary Methods**

**Plasmid constructions.** Plasmids pT7-CdsA and pT7-YnbB carrying *cdsA* and *ynbB*, respectively, under the control of the T7 promoter, were constructed as follows. The *cdsA* and *ynbB* genes were PCR-amplified using a pair of primers (cdsA-5´ Nde and cdsA-3´ Sal) and a pair of primers (ynbB-5´ Nde and ynbB-3´ Sal), and chromosomal DNA prepared from JM109. The amplified fragments were treated with NdeI and SalI, followed by cloning into the same sites of pIVEX2.4b-Nde. The DNA fragments containing *cdsA* and *ynbB* were then obtained by cutting with NdeI and XhoI, followed by cloning into the same sites of pET-15b, yielding pT7-CdsA and pT7-YnbB, respectively.

Plasmid pAra-CdsA, in which *cdsA* is under the control of the *ara* regulon, was constructed as follows. The *cdsA* gene was PCR-amplified using a pair of primers (cdsA-5´ and cdsA-3´ comp) and then treated with BamHI and SalI, followed by cloning into the same sites of pUSI2, yielding pTac-CdsA. Then, the DNA fragment of *cdsA*, obtained by cutting pTac-CdsA with BamHI and BglII, was cloned into the BglII site of pKQ2, yielding pKQ2-CdsA. The *bla* gene on pKQ2-CdsA was replaced with the *spc* gene, yielding pAra-CdsA. This was achieved by ligating the fragment PCR-amplified using a pair of primers (pKQ2 up comp and pKQ2 down) and pKQ2-CdsA as temperate, with the *spc* fragment PCR-amplified using a pair of primers (spc-5´ and spc-3´ comp) and pHP45Ω as temperate. Plasmid pTac-CdsA8 carries the *cdsA8* allele instead of the *cdsA* allele on pTac-CdsA.

Plasmids pTac-Tam41p and pTet-Tam41p carrying *TAM41* without mitochondrial targeting signal under the control of the *tac* promoter/*lac* operator and under the *tet* promoter, respectively, were constructed as follows. The *TAM41* DNA fragment PCR-amplified using a pair of primers (TAM41-5´ and TAM41-3´ comp) and RKP153 as temperate, was treated with BamHI and SalI, and then cloned into the same sites of pUSI2, yielding pTac-Tam41p. Plasmid pACYC-Km was constructed so that the *cat* gene on plasmid pACYC184 was replaced with the *kan* gene. The *kan* gene fragment amplified using a pair of primers (kan-5´ and kan-3´ comp) and pUC4K as temperate, was ligated to the vector fragment amplified using a pair of primers (pACYC up comp and pACYC down) and pACYC184 as temperate, after both fragments were treated with BglII and EcoRI. The *TAM41* DNA fragment treated with BamHI and SalI was cloned into the same sites of pACYC-Km, yielding pTet-Tam41p.

Plasmids pTet-CdsA and pTet-CdsA8, which carry *cdsA* and *cdsA8* under the control of the *tet* promoter, respectively, were constructed by ligating the BamHI-SalI fragments of pTac-CdsA and pTac-CdsA8 with pACYC-Km cut with BamHI and SalI.

Plasmid pTet-Cds1p-Hs, which encodes *Hs-CDS1* under the control of the *tet* promoter, was constructed as follows. A chemically synthesized DNA fragment of *Hs-CDS1* (1-945 bp) with attached restriction sites of BamHI at the 5´ end and HpaI-KpnI at the 3´ end, respectively, was digested with BamHI and KpnI, and then cloned into the same sites of pUC118, yielding pUC118-CDS1 (1-945). Another chemically synthesized DNA fragment of *Hs-CDS1* (946-1386 bp) with attached restriction sites of BamHI-HpaI at the 5´ end and KpnI-BglII at the 3´ end, respectively, was digested BamHI and KpnI, and then cloned into the same sites of pUC118, yielding pUC118-CDS1 (946-1386). A gene fragment of *Hs-CDS1* (946-1386 bp), obtained by digestion with HpaI and KpnI, was then ligated into the same sites of pUC118-CDS1 (1-945), yielding pUC118-CDS1 carrying the entire *Hs-CDS1*. A gene fragment encoding Hs-Cds1p, obtained by digesting pUC118-CDS1 with BamHI and KpnI, was cloned into the same sites of pUSI2, yielding pTac-Cds1p-Hs. The gene fragment encoding Hs-Cds1p, obtained by digesting pTac-Cds1p-Hs with BamHI and SalI, was cloned into the same sites of pACYC-Km, yielding pTet-Cds1p-Hs.

**Chemical synthesis of compound I and CDP-GlcNAc.** All chemicals were reagent grade and purchased from Nacalai tesque. Unless otherwise noted, all chemicals and solvents were used as supplied. Moisture sensitive reactions were performed under argon atmosphere. All reactions were monitored by TLC on Silica Gel 60F_254_ precoated glass slides (Merck). Detection was performed by examination under UV light (254 nm) and/or by charring with 5% 12molybdo(VI)phosphoric acid in EtOH. Flash column chromatography was performed on silica gel (Nacalai, Silica Gel 60 mesh 230-400 or spherical). ^1^H, ^13^C, ^31^P NMR spectra were recorded at 298 K on Bruker AVANCE III HD-400 spectrometer and analyzed with solvent peaks as internal references or 85% H_3_PO_4_ as external standard. High-resolution ESI-MS was measured on Shimadzu LC-IT-TOF-MS spectrometer with CH_3_CN /10 mM aqueous AcONH_4_ or acetone /10 mM aqueous AcONH_4_ as the mobile phase and sodium trifluoroacetate as an external standard.

**Supplementary Fig. 18. Synthesis of Compound I**

**2-acetamido-3,4,6-tri-*O*-benzyl-2-deoxyglucopyranose 1-*O*-diallylphosphate (2).** To a solution of 2-acetamido-3,4,6-tri-*O*-benzyl-2-deoxyglucopyranose (**1**) (390 mg, 0.79 mmol) in CH_2_Cl_2_, diallyl *N,N*-diisopropylphosphoramidite (635 μL, 2.38 mmol) and 1H-tetrazole (165 mg, 2.38 mmol) were added at room temperature. The reaction mixture was stirred for 1 h followed by the addition of *tert*-butyl hydroperoxide (800 μL). After 1 h, the reaction was quenched with aq. Na_2_S_2_O_3_, which was extracted with AcOEt. The combined organic phase was washed with brine and dried over MgSO_4_. The obtained crude product was purified by flash column chromatography (hexane : AcOEt = 1 : 1 → 2 : 1→ AcOEt only) to give the desired product **2** (303 mg, 59%). ^1^H NMR (400 MHz, CDCl_3_) δ 7.15-7.45 (15 H, m), 5.87 (2 H, m), 5.66 (1 H, dd, *J* = 3.8, 6.2 Hz), 5.42 (1 H, m), 5.32 (2 H, d, *J* = 17.3 Hz), 5.22 (2 H, dd, *J* = 3.8, 10.2 Hz), 4.85 (1 H, d, *J* = 10.9 Hz), 4.83 (1 H, d, *J* = 10.4 Hz), 4.64 (1 H, d, *J* = 10.9 Hz), 4.60 (1 H, d, *J* = 11.5 Hz), 4.58 (1 H, d, *J* = 10.4 Hz), 4.52 (4 H, m), 4.49 (1 H, d, *J* = 11.5 Hz), 4.30 (1 H, dd, *J* = 10.0, 10.0 Hz), 3.99 (1 H, d, *J* = 10.0 Hz), 3.81 (1 H, dd, *J* = 9.5, 9.5 Hz), 3.76 (1 H, dd, *J* = 3.5, 10.0 Hz), 3.70 (1 H, dd, *J* = 9.5, 10.0 Hz), 3.65 (1 H, d, *J* = 10.0 Hz), 1.81 (3 H, s). ^13^C-NMR (100 MHz, CDCl_3_) δ 170.0, 138.10, 137.82, 132.30, 132.23, 132.07, 132.00, 128.57, 128.48, 128.39, 128.25, 127.96, 127.89, 127.78, 127.71, 118.78, 118.53, 97.48, 97.41, 78.87, 77.77, 75.12, 74.87, 73.46, 73.11, 68.56, 68.52, 68.47, 68.04, 52.54, 52.47, 23.18. ^31^P-NMR (162 MHz, CDCl_3_) δ -2.38. HRMS for C_35_H_42_NNaO_9_P [M+Na^+^] found 674.2490, calcd 674.2489.

**2-acetamido-3,4,6-tri-*O*-benzyl-2-deoxyglucopyranose 1-*O*-phosphate (3).** To a solution of **2** (303 mg, 0.46 mmol) in THF, tetrakis(triphenylphosphine)palladium(0) (107 mg, 0.093 mmol) and diethylmine (970 μL, 9.3 mmol) were added at room temperature. The reaction mixture was stirred for 1 h. The mixture was concentrated and purified by flash column chromatography (MeOH : CHCl_3_ : Et_3_N = 5 : 95 : 1 → 20 : 80 : 1→ 50 : 50 : 1) to give the desired product **3** (287 mg, 93%). ^1^H NMR (400 MHz, CDCl_3_) δ 7.15-7.35 (15 H, m), 5.47 (1 H, d, *J* = 7.3 Hz), 4.78 (1 H, d, *J* = 11.0 Hz), 4.75 (1 H, d, *J* = 10.5 Hz), 4.73 (1 H, d, *J* = 10.5 Hz), 4.50 (1 H, d, *J* = 11.0 Hz), 4.49 (1 H, d, *J* = 12.0 Hz), 4.39 (1 H, d, *J* = 12.0 Hz), 4.35 (1 H, dd, *J* = 10.5, 10.5 Hz), 4.09 (1 H, d, *J* = 11.2 Hz), 3.88 (1 H, dd, *J* = 10.0 Hz), 3.67 (1 H, dd, *J* = 8.5, 8.5 Hz), 3.62 (2 H, m), 2.83 (6 H, q, *J* = 7.2 Hz), 2.64 (1 H, bs), 1.95 (3 H, s), 1.16 (9 H, t, *J* = 7.2 Hz). ^13^C-NMR (100 MHz, CDCl_3_) δ171.05, 138.72, 138.49, 138.06, 128.31, 128.27, 128.24, 127.89, 127.89, 127.86, 127.62, 127.53, 127.41, 94.44, 80.99, 78.06, 75.03, 74.76, 73.33, 71.65, 69.33, 53.29, 53.24, 45.38, 23.33, 8.74. ^31^P-NMR (162 MHz, CDCl_3_) δ -0.19**.** HRMS for C_29_H_33_NO_9_P [M-H^-^] found 570.1869, calcd 570.1898.

**(2R)-3-(benzyloxy(diisopropylamino)phosphinooxy)propane-1,2-diyl dipalmitate (5).** To a solution of 1,2-dipalmitoyl-*sn*-glycerol (**4**) (71 mg, 0.125 mmol) and benzyl N,N,N´,N´-tetraisopropylphosphoramidite^66^ (85 mg, 0.25 mmol) in CH_2_Cl_2_, 1H-tetrazole (165 mg, 2.38 mmol) was added at room temperature and the reaction mixture was stirred for 20 min. The mixture was concentrated and purified by flash column chromatography (hexane : AcOEt : Et_3_N = 80 : 10 : 2) to give the desired product **5** (90 mg, 90%). ^1^H NMR (400 MHz, CDCl_3_) δ 7.45-7.20 (5 H, m), 5.20 (1 H, m), 4.80-4.62 (2 H, m), 4.35 (1 H, dd, *J* = 3.1, 11.1 Hz), 4.18 (1 H, dd, *J* = 5.6, 11.1 Hz), 3.78 (1 H, m), 3.72 (1 H, m), 3.64 (2 H, m), 2.29 (4 H, t, *J* = 7.5 Hz), 1.61 (4 H, m), 1.26 (48 H, m), 1.19 (6 H, d, *J* = 6.5 Hz), 1.18 (6 H, d, *J* = 6.5 Hz), 0.89 (6 H, d, *J* = 6.5 Hz). ^13^C-NMR (100 MHz, CDCl_3_) δ173.39, 173.01, 128.24, 128.20, 127.27, 126.93, 70.85, 70.80, 70.73, 65.45, 65.27, 62.51, 62.47, 61.66, 43.13, 43.01, 34.33, 34.13, 31.92, 29.69, 29.66, 29.48, 29.35, 29.29, 29.13, 29.12, 24.90, 24.67, 24.59, 24.51, 22.68, 14.10. ^31^P-NMR (162 MHz, CDCl_3_) δ 148.99, 148.83.

**(2R)-3-((2-acetamido-3,4,6-tri-*O*-benzyl-2-deoxyglucopyranosyloxy(hydroxy)-phosphoryloxy)(benzyloxy)phosphoryloxy)propane-1,2-diyl dipalmitate (6).** To a solution of **3** (28 mg, 0.042 mmol) and **4** (38 mg, 0.047 mmol) in CH_2_Cl_2_, dicyanoimidazole (5.5 mg, 0.047 mmol) was added at room temperature. The reaction mixture was stirred for 20 min prior to the addition of 5 M tert-butyl hydroperoxide in decane (50 μL). The mixture was purified by flash column chromatography (MeOH : CHCl_3_ : Et_3_N = 90 : 10 : 1) to give the desired product **6** (38 mg, 66%). ^1^H NMR (400 MHz, CDCl_3_) δ 7.15-7.55 (20 H, m), 5.58 (1 H, d, *J* = 6.0 Hz), 5.22 (1 H, m), 5.17 (1 H, m), 5.12 (1 H, m), 4.82 (1 H, d, *J* = 10.9 Hz), 4.71 (2 H, m), 4.53 (1 H, d, *J* = 10.9 Hz), 4.51 (1 H, d, *J* = 10.9 Hz), 4.44 (1 H, d, *J* = 10.9 Hz), 4.41 (1 H, m), 4.33 (1 H, m), 4.30 (1 H, m), 4.19 (1 H, m), 4.15 (1 H, m), 4.11 (1 H, m), 3.86 (1 H, dd, *J* = 11.0, 11.0 Hz), 3.73 (1 H, dd, *J* = 9.7, 9.7Hz), 3.70 (1 H, m), 3.61 (1 H, m), 2.90 (6 H, q, *J* = 7.5 Hz), 2.25 (4 H, m), 1.95 (3/2 H, s), 1.92 (3/2 H, s), 1.55 (4 H, m), 1.32-1.17 (59 H, m), 0.88 (6 H, t, *J* = 6.5 Hz). ^13^C-NMR (100 MHz, CDCl_3_) δ 173.27, 172.85, 170.89, 138.57, 138.15, 128.52, 128.42, 128.29, 127.88, 127.80, 127.70, 127.54, 127.47, 127.42, 96.30, 80.78, 77.93, 75.26, 74.75, 72.12, 69.79, 69.74, 69.43, 69.04, 65.96, 62.01, 61.88, 52.93, 45.86, 31.91, 29.69, 29.49, 29.35, 29.29, 29.12, 24.81, 22.67, 14.10, 9.33. ^31^P-NMR (162 MHz, CDCl_3_) δ -11.76 (d, J = 17.5 Hz), -12.00 (d, J = 17.5 Hz), -13.17 (d, J = 17.5 Hz), -13.33 (d, J = 17.5 Hz). HRMS for C_71_H_106_NO_16_P_2_ [M-H]^-^ found 1290.7080, calcd. 1290.6992.

**(2R)-3-((2-acetamido-2-deoxyglucopyranosyloxy(hydroxy)phosphoryloxy)-(hydroxy)phosphoryloxy)propane-1,2-diyl dipalmitate (Compound I).** A solution of **6** (49 mg, 0.035 mmol) in MeOH / THF (1/1) was hydrogenated in the presence of Pd-black (10 mg) for 2 h. The suspension was filtered and concentrated *in vacuo* to give the titled product (**7)** (25 mg, 69%). ^1^H NMR (400 MHz, CD_3_OD) δ 5.52 (1 H, dd, *J* = 3.1, 7.1 Hz), 5.26 (1 H, m), 4.45 (1 H, dd, *J* = 2.6, 12.0 Hz), 4.19 (1 H, dd, *J* = 6.9, 12.0 Hz), 4.15 (2 H, m), 3.98 (1 H, m), 3.92 (1 H, m), 3.83 (1 H, dd, *J* = 1.7, 12.0 Hz), 3.73 (1 H, dd, *J* = 10.0, 10.0 Hz), 3.68 (1 H, dd, *J* = 5.8, 12.0 Hz), 3.19 (12 H, q, *J* = 7.3 Hz), 2.32 (2 H, t, *J* = 7.6 Hz), 2.30 (2 H, t, *J* = 7.6 Hz), 2.05 (3 H, s), 1.59 (4 H, m), 1.34-1.26 (66 H, m), 0.89 (6 H, t, *J* = 7.0 Hz). ^13^C NMR (100 MHz, CD_3_OD) δ 172.24, 171.97, 171.82, 93.80, 72.32, 70.65, 69.34, 62.67, 61.31, 60.14, 52.82, 44.98, 32.47, 32.26, 30.40, 28.00, 27.56, 23.35, 21.06, 20.33, 11.76, 6.47. ^31^P NMR (162 MHz, CD_3_OD) δ-12.55 (d, *J* = 22.5 Hz), -14.38 (d, *J* = 22.5 Hz). HRMS for C_43_H_82_NO_16_P_2_ [M-H]^-^ found 930.5125, calcd. 930.5114.

**Supplementary Fig. 19. Synthesis of CDP-GlcNAc**

**2-acetamido-3,4,6-tri-*O*-acetyl-2-deoxyglucopyranose 1-*O*-dibenzylphosphate (8).** To a solution of 2-acetamido-3,4,6-tri-*O*-acetyl-2-deoxyglucopyranose (**7**) (270 mg, 0.78 mmol) in CH_2_Cl_2_, dibenzyl *N,N*-diisopropylphosphoramidite (540 mg, 2.33 mmol) and 1H-tetrazole (163 mg, 2.33 mmol) were added at -20°C. The reaction mixture was stirred at room temperature for 1.5 h followed by the addition of 5 M tert-butyl hydroperoxide in decane (460 μL) at -40°C. After 1 h, the reaction was quenched with aq. Na_2_S_2_O_3_, which was extracted with AcOEt. The combined organic phase was washed with brine and dried over MgSO_4_. The obtained crude product was purified by flash column chromatography (hexane : AcOEt = 2 : 1→ AcOEt only) to give the desired product **8** (329 mg, 69%). ^1^H NMR (400 MHz, CDCl_3_) δ 7.42-7.30 (10 H, m), 5.75 (1 H, d, *J* = 9.2 Hz), 5.66 (1 H, dd, *J* = 3.3, 6.0 Hz), 5.16 (1 H, m), 5.14 (1 H, m), 5.07 (4 H, m), 4.36 (1 H, ddd, *J* = 3.3, 9.2, 9.5 Hz), 4.12 (1 H, dd, *J* = 3.7, 12.2 Hz), 3.99 (1 H, m), 3.91 (1 H, dd, *J* = 1.7, 12.2 Hz), 2.02 (3 H, s), 2.00 (6 H, s), 1.70 (3 H, s). ^13^C-NMR (100 MHz, CDCl_3_) δ 171.09, 170.50, 170.15, 163.07, 135.32, 135.25, 135.17, 135.11, 128.91, 128.78, 128.75, 128.07, 128.02, 96.22, 96.15, 70.03, 69.95, 69.90, 69.85, 69.57, 67.26, 61.20, 51.75, 51.67, 22.69, 20.60, 20.57, 20.50. ^31^P-NMR (162 MHz, CDCl_3_) δ −2.30.

**2-acetamido-3,4,6-tri-*O*-acetyl-2-deoxyglucopyranose 1-*O*-phosphate (9).** A solution of **8** (329 mg, 0.54 mmol) in EtOH was hydrogenated in the presence of Pd-black (30 mg) for 3 h. The suspension was filtered and concentrated *in vacuo* to give the titled product (**9)** (228 mg, quant.). ^1^H NMR (400 MHz, CDCl_3_) δ 10.66 (2 H, bs), 7.62 (1 H, bs), 5.71 (1 H, m), 5.30 (1 H, dd, *J* = 9.5, 10.0 Hz), 5.15 (1 H, dd, *J* = 9.5, 10.0 Hz), 4.34 (1 H, m), 4.28 (1 H, m), 4.25 (1 H, m), 4.19 (1 H, m), 2.08 (6 H, s), 2.02 (3 H, s), 2.01 (3 H, s). ^13^C NMR (100 MHz, CDCl_3_) δ 174.27, 171.11, 170.99, 169.41, 94.21, 69.86, 69.06, 67.82, 61.44, 52.41, 21.93, 20.67, 20.62, 20.48. ^31^P NMR (162 MHz, CD_3_OD) δ -2.11. HRMS for C_14_H_21_NO_12_P [M-H]^-^ found 426.0803, calcd. 426.0807.

**4-*N*-acetyl-2´,3´-di-*O*-acetyl-5´-*O*-((4-acetoxybenzyloxy)(diisopropylamino)-phosphino)cytidine (11).** To a solution of 4-*N*-acetyl-2´,3´-di-*O*-acetylcytidine^67^ (**10**) (37 mg, 0.10 mmol) and 4-acetoxybenzyl N,N,N´,N´-tetraisopropylphosphoramidite^66^ (80 mg, 0.20 mmol) in CH_2_Cl_2_, 1H-tetrazole (7.0 mg, 0.10 mmol) was added at 0 °C and the reaction mixture was stirred for 2 h. The reaction was quenched with aq. NaHCO_3_, which was extracted with AcOEt. The combined organic phase was washed with brine and dried over MgSO_4_. The obtained crude product was purified by flash column chromatography (hexane : AcOEt : Et_3_N = 67 : 33 : 3 → AcOEt : Et_3_N = 100 : 3) to give the desired product **11** (49 mg, 74%). ^1^H NMR (400 MHz, CDCl_3_) δ 9.50 (1/2 H, bs), 9.40 (1/2 H, bs), 8.25 (1/2 H, d, *J* = 7.6 Hz), 8.25 (1/2 H, d, *J* = 7.6 Hz), 7.36 (1 H, dd, *J* = 7.5 Hz), 7.34 (1 H, dd, *J* = 7.5 Hz), 7.06 (1 H, dd, *J* = 7.5 Hz), 7.04 (1 H, dd, *J* = 7.5 Hz), 6.36 (1/2 H, d, *J* = 6.1 Hz), 6.34 (1/2 H, d, *J* = 5.2 Hz), 5.46 (1 H, m), 5.39 (1 H, m), 5.38 (1 H, m), 4.77 (1 H, m), 4.67 (1 H, m), 4.33 (1 H, m), 3.98 (1/2 H, m), 3.87 (1 H, m), 3.85 (1/2 H, m), 3.66 (2 H, m), 2.282 (3/2 H, s), 2.279 (3/2 H, s), 2.24 (2/3 H, s), 2.23 (3/2 H, s), 2.10 (3 H, s), 2.054 (3/2 H, s), 2.048 (3/2 H, s), 1.21 (12 H, d, *J* = 6.5 Hz). ^31^P NMR (162 MHz, CDCl_3_) δ 149.31, 149.13.

**4-*N*-acetyl-2´,3´-di-*O*-acetyl-5´-*O*-((2-acetamido-3,4,6-tri-*O*-acetyl-2-deoxygluco-pyranosyloxy(hydroxy)phosphoryloxy)(4-acetoxybenzyloxy)phosphoryl)cytidine (12).** To a solution of **9** (68 mg, 0.13 mmol) and **11** (130 mg, 0.20 mmol) in CH_3_CN, dicyanoimidazole (24 mg, 0.20 mmol) in CH_3_CN was added dropwise at room temperature. The reaction mixture was stirred for 20 min prior to the addition of 5 M tert-butyl hydroperoxide in decane (100 μL). The mixture was purified by flash column chromatography (MeOH : CHCl_3_ : Et_3_N = 99 : 1 : 3 → 90 : 10 : 3) to give the desired product **12** as Et_3_N salt (72 mg, 50%). ^1^H NMR (400 MHz, CDCl_3_) δ 9.36 (1 H, bs), 8.29 (2/3 H, d, *J* = 7.6 Hz), 8.10 (1/3 H, d, *J* = 6.8 Hz), 7.44 (2/3 H, d, *J* = 7.2 Hz), 7.40 (4/3 H, d, *J* = 8.5 Hz), 7.09-7.03 (2 H, m), 6.24 (1 H, d, J = 4.4 Hz), 5.63 (1 H, dd, J = 2.9, 7.1 Hz), 5.38 (1 H, m), 5.37 (1 H, m), 5.30 (1 H, dd, *J* = 9.8, 10.0 Hz), 5.20 (1 H, m), 5.18 (1 H, m), 5.14 (2 H, m), 4.59 (1 H, m), 4.41 (1 H, m), 4.39 (1 H, m), 4.39-4.30 (2 H, m), 4.24 (1 H, m), 4.16-4.05 (2 H, m), 3.67 (12 H, q, *J* = 7.2 Hz), 2.28 (3 H, s), 2.22 (1 H, s), 2.21 (2 H, s), 2.071 (3 H, s), 2.068 (3 H, s), 2.06 (3 H, s), 2.00 (1 H, s), 1.99 (1 H, s), 1.98 (4 H, s), 1.96 (2 H, s), 1.94 (1 H, s), 1.35 (18 H, t, *J* = 7.2 Hz). ^13^C NMR (100 MHz, CDCl_3_) δ 171.14, 170.76, 170.55, 169.63, 169.50, 169.43, 169.39, 169.31, 162.70, 155.33, 150.78, 133.12, 129.30, 129.13, 121.88, 121.83, 97.38, 95.53, 95.46, 87.70, 80.67, 80.58, 73.74, 70.92, 69.61, 69.11, 69.07, 68.79, 67.99, 66.27, 61.22, 51.57, 51.52, 45.79, 25.00, 22.75, 21.07, 20.73, 20.66, 20.59, 20.45, 20.44, 8.61. ^31^P NMR (162 MHz, CDCl_3_) δ -11.21, -11.32, -13.67, -13.78. HRMS for C_38_H_47_N_4_O_24_P_2_ [M-H]^-^ found 1005.2055, calcd. 1005.2061.

**5´-*O*-((2-acetamido-2-deoxyglucopyranosyloxy(hydroxy)phosphoryloxy)(hydroxy)-phosphoryloxy)cytidine (CDP-GlcNAc).** A solution of **12** (22 mg, 0.020 mmol) in Et_3_N / MeOH / H_2_O (1 / 7 / 3) was stirred at room temperature for 18 h. The mixture was concentrated and purified by gel-chromatography (LH-20, H_2_O) followed by lyophilization to give the desired product **CDP-GlcNAc** as Et_3_N salt (9.4 mg, 67%). ^1^H NMR (400 MHz, D_2_O) δ 8.03 (1 H, d, J = 7.5 Hz), 6.17 (1 H, d, *J* = 7.5 Hz), 6.00 (1 H, d, *J* = 3.7 Hz), 5.54 (1 H, dd, *J* = 2.8, 7.1 Hz), 4.37 (1 H, m), 4.34 (1 H, m), 4.31 (1 H, m), 4.29 (1 H, m), 4.23 (1 H, m), 4.01 (1 H, ddd, *J* = 3.2, 3.2, 10.5 Hz), 3.95 (1 H, ddd, *J* = 1.9, 4.0, 10.5 Hz), 3.88 (1 H, dd, *J* = 2.1, 12.2 Hz), 3.84 (1 H, m), 3.83 (1 H, m), 3.57 (1 H, dd, *J* = 9.5 9.5 Hz), 3.22 (12 H, q, *J* = 7.2 Hz), 2.09 (3 H, s), 1.30 (18 H, t, *J* = 7.2 Hz). ^13^C NMR (100 MHz, D_2_O) δ 174.72, 165.04, 141.80, 96.32, 94.48, 94.43, 89.28, 74.22, 72.95, 70.89, 69.43, 69.20, 64.63, 60.24, 53.65, 53.58, 46.62, 22.00, 8.16. ^31^P NMR (162 MHz, CDCl_3_) δ -11.23 (d, *J* = 20.5 Hz), -12.97 (d, *J* = 20.5 Hz). HRMS for C_17_H_27_N_4_O_16_P_2_ [M-H]^-^ found 605.0827, calcd. 605.0903.

**Supplementary References**

55. Yanisch-Perron, C., Vieira, J. & Messing, J. Improved M13 phage cloning vectors and host strains: nucleotide sequences of the M13mp18 and pUC19 vectors. *Gene* **33**, 103-119 (1985).

56. Stahl, F.W., Kobayashi, I., Thaler, D. & Stahl, M.M. Direction of travel of RecBC recombinase through bacteriophage lambda DNA. *Genetics* **113**, 215-227 (1986).

57. Chen, M., Xie, K., Nouwen, N., Driessen, A.J. & Dalbey, R.E. Conditional lethal mutations separate the M13 procoat and Pf3 coat functions of YidC: different YIDC structural requirements for membrane protein insertion. *J. Biol. Chem.* **278**, 23295-23300 (2003).

58. Inaba, K. *et al.* Crystal structure of the DsbB-DsbA complex reveals a mechanism of disulfide bond generation. *Cell* **127**, 789-801 (2006).

59. Nishiyama, K., Hanada, M. & Tokuda, H. Disruption of the gene encoding p12 (SecG) reveals the direct involvement and important function of SecG in the protein translocation of *Escherichia coli* at low temperature. *EMBO J.* **13**, 3272-3277 (1994).

60. Prentki, P. & Krisch, H.M. *In vitro* insertional mutagenesis with a selectable DNA fragment. *Gene* **29**, 303-313 (1984).

61. Chang, A.C. & Cohen, S.N. Construction and characterization of amplifiable multicopy DNA cloning vehicles derived from the P15A cryptic miniplasmid. *J. Bacteriol.* **134**, 1141-1156 (1978).

62. Vieira, J. & Messing, J. The pUC plasmids, an M13mp7-derived system for insertion mutagenesis and sequencing with synthetic universal primers. *Gene* **19**, 259-268 (1982).

63. Kiefer, D. & Kuhn, A. Hydrophobic forces drive spontaneous membrane insertion of the bacteriophage Pf3 coat protein without topological control. *EMBO J.* **18**, 6299-6306 (1999).

64. Nishiyama, K. & Tokuda, H. Preparation of a highly translocation-competent proOmpA/SecB complex. *Protein Sci.* **19**, 2402-2408 (2010).

65. Vieira, J. & Messing, J. Production of single-stranded plasmid DNA. *Methods Enzymol.* **153**, 3-11 (1987).

66. Weinschenk, L., Schols, D., Balzarini, J. & Meier, C. Nucleoside diphosphate prodrugs: nonsymmetric DiPPro-nucleotides. *J. Med. Chem.* **58**, 6114-6130 (2015).

67. Chappell, M.D. & Halcomb, R.L. Synthesis of CMP-sialic acid conjugates: substrates for the enzymatic synthesis of natural and designed sialyl oligosaccharides. *Tetrahedron* **53**, 11109-11120 (1997).
